# The Set3/Hos2 Histone Deacetylase Complex Attenuates cAMP/PKA Signaling to Regulate Morphogenesis and Virulence of *Candida albicans*

**DOI:** 10.1371/journal.ppat.1000889

**Published:** 2010-05-13

**Authors:** Denes Hnisz, Olivia Majer, Ingrid E. Frohner, Vukoslav Komnenovic, Karl Kuchler

**Affiliations:** 1 Medical University Vienna, Christian Doppler Laboratory for Infection Biology, Max F. Perutz Laboratories, Vienna, Austria; 2 Institute of Molecular Biotechnology of the Austrian Academy of Sciences, Vienna, Austria; University of Toronto, Canada

## Abstract

*Candida albicans*, like other pleiomorphic fungal pathogens, is able to undergo a reversible transition between single yeast-like cells and multicellular filaments. This morphogenetic process has long been considered as a key fungal virulence factor. Here, we identify the evolutionarily conserved Set3/Hos2 histone deacetylase complex (Set3C) as a crucial repressor of the yeast-to-filament transition. Cells lacking core components of the Set3C are able to maintain all developmental phases, but are hypersusceptible to filamentation-inducing signals, because of a hyperactive cAMP/Protein Kinase A signaling pathway. Strikingly, Set3C-mediated control of filamentation is required for virulence *in vivo*, since *set3*Δ/Δ cells display strongly attenuated virulence in a mouse model of systemic infection. Importantly, the inhibition of histone deacetylase activity by trichostatin A exclusively phenocopies the absence of a functional Set3C, but not of any other histone deacetylase gene. Hence, our work supports a paradigm for manipulating morphogenesis in *C. albicans* through alternative antifungal therapeutic strategies.

## Author Summary

*Candida albicans* is the most prevalent human fungal pathogen causing disease in immunocompromised individuals. One key virulence factor, shared by many other microbial pathogens, is its ability to undergo morphogenetic transitions between unicellular and filamentous forms, which interact differentially with the host immune system. Morphogenesis in *C. albicans* is controlled by several signaling pathways, of which the Protein Kinase A (PKA) nutrient sensing pathway is of pivotal importance. Here, we identify a role for a conserved histone deacetylase complex (Set3C) as a key negative regulator of PKA signaling, controlling both morphogenesis and virulence. The genetic removal of the Set3C histone deacetylase complex causes cells to hyperfilament both *in vitro* and *in vivo*, and triggers the activation of PKA signaling. Moreover, cells lacking Set3 show strongly attenuated virulence in a mouse infection model. These results provide novel insights about the interplay of chromatin modification and signaling pathways, as exemplified by the control of morphogenesis of pleiomorphic fungi. Furthermore, our work establishes histone deacetylases as potential novel targets for antifungal drug discovery to improve therapeutic approaches combating life-threatening fungal infections.

## Introduction

The human fungal pathogen *Candida albicans* is a harmless commensal of the mucosal surfaces and gastrointestinal tract of most healthy individuals. However, it can cause severe superficial and disseminated infections, particularly when the immune system of the human host is compromised [1]. A major virulence trait of *C. albicans* is the ability to switch between several distinct morphologies, including the unicellular yeast-like and the filamentous pseudohyphal and hyphal forms. Since several other fungal pathogens are also dimorphic or even pleiomorphic, including *Blastomyces dermatitidis* or *Coccidioides immiti*, morphogenesis has been considered a key component of fungal virulence and host invasion [2], [3].

The switch from the yeast to filamentous forms in *C. albicans* is triggered by a broad range of environmental or host stimuli, including serum, an elevated growth temperature to 37°C *in vitro*, and more specific inducers such as N-acetylglucosamine or estradiol [4], [5]. The control of this morphological transition involves several signaling pathways and transcription factors [6], [7]. The most prominent positive regulators are a mitogen-activated protein (MAP) kinase pathway and its downstream transcription factor Cph1 [8], as well as the cyclic adenosine monophosphate (cAMP)/protein kinase A (PKA) pathway and its downstream target transcription factor Efg1 [9], [10]. In addition, filamentation is also under negative control by the transcriptional repressor Tup1 [11]. Tup1-mediated repression requires an interaction between Tup1 with sequence-specific DNA-binding factors such as Nrg1 and Rfg1 [12], [13], [14].

In addition to the yeast-filament transition, diploid *C. albicans* cells homozygous for the Mating Type Locus (*MTL*) can also reversibly switch between two distinct cell types termed white and opaque. White cells have a round, yeast-like shape and form dome-shaped colonies on solid agar, while opaque cells display an elongated morphology and form flattened colonies [15]. Furthermore, white cells are unable to mate, whereas opaque cells are mating-competent [16]. Switching between the two phases is believed to enable *C. albicans* to better adapt to various host niches. For example, white cells are more virulent in a murine systemic infection model, whereas opaque cells colonize the skin more efficiently than white cells [17], [18].

White-opaque switching is reversible and regulated by several transcription factors. The *C. albicans* diploid genome harbors the Mating Type Locus (*MTL*) with two alleles known as **a** and α [19]. The master regulator of switching is *WOR1*, which in *MTL* heterozygous cells is stably repressed by a heterodimeric **a**/α repressor [16], [20], [21]. *MTL* homozygous white cells are devoid of detectable Wor1 expression. By contrast, opaque cells maintain high Wor1 levels for many generations due to multiple positive feed-back loops [22]. Interestingly, *EFG1* is a key negative regulator of *WOR1*, since *MTL* homozygous *efg1*Δ/Δ cells exist predominantly in the opaque phase [23], [24].

Recently, we have shown that several histone-modifier enzymes, including the evolutionarily conserved Set3/Hos2 histone deacetylase complex (SetC) also modulate the white-opaque transition [25]. The related Set3 and Hos2 proteins in *Saccharomyces cerevisiae* are parts of a similar multiprotein complex (Set3C) that possesses histone deacetylase activity, with multiple functions, including meiosis-specific repression of sporulation [26], promotion of Ty1 retrotransposons integration at tRNA genes [27], as well as signaling secretory stress through the PKC cell integrity pathway [28].

In this study, we discover a unique and novel composite phenotype caused by the deletions of key subunits of the Set3/Hos2 complex in *C. albicans*. On solid media, *set3*Δ/Δ and *hos2*Δ/Δ mutants display a hyperfilamenous phenotype at elevated temperatures, and specifically in the opaque phase. Surprisingly, in the filamentous forms of *set3*Δ/Δ and *hos2*Δ/Δ cells, Efg1-dependent target genes are induced as a result of an activated cAMP/PKA pathway. Our results establish the Set3C as a PKA-antagonist that fine-tunes the threshold of the yeast-filament conversion. We also show that *set3*Δ/Δ cells display strongly attenuated virulence in a murine model of systemic infection, which is associated with hyperfilamentation *in vivo*. Strikingly, we demonstrate that inhibition of histone deacetylation by trichostatin A exclusively phenocopies the lack of Set3C but not of any other histone deacetylase in *Candida albicans.* Hence, these data suggest a novel approach for developing antifungal drugs by manipulating the morphogenetic ability of *Candida albicans* through inhibition of chromatin modification.

## Results

### *MTL* homozygous *set3*Δ/Δ mutants filament specifically in the opaque phase

The Set3 histone deacetylase complex (Set3C) of *Saccharomyces cerevisiae* comprises of four core (Set3, Hos2, Snt1, Sif2) and three peripheral subunits (Hos4, Hst1, Cpr1), of which Hos2 and Hst1 display catalytic activities. Disruption of any of the core subunit genes prevents complex assembly, whereas the disruption of peripheral subunit genes has no such effect [26]. Reciprocal BLAST searches of the *S. cerevisiae* and *C. albicans* proteomes revealed a strong conservation of the core proteins and suggested an analogous architecture of the putative CaSet3C (Table 1).

**Table 1:** Components of the *S. cerevisiae* and *C. albicans* Set3 Complex components.

*S.c*. Gene	*S.c.* Systematic name	Required for structural integrity (S.c.)	*C.a*. ORF	Length (*C.a*., aa)	Homology value (aa)
*SET3*	YKR029C	+	19.7221	1069	2.00E-47
*HOS2*	YGL194C	+	19.5377	454	<1E-150
*SNT1*	YCR033W	+	19.5241	1001	6.00E-37
*SIF2*	YBR103W	+	19.132	593	1.00E-34
*HOS4*	YIL112W	–	19.4728	1380	4.00E-31
*HST1*	YOL068C	–	19.4761	657	5.00E-105
*CPR1*	YDR155C	–	19.6472	162	1.00E-73

In the absence of the subunits required for structural integrity, no complex can assemble in *S. cerevisiae* [26]. The BLAST E-values of the protein sequences indicate that every subunit is evolutionary conserved (E-value <10^−30^) in *C. albicans*. “aa”: amino acid.

**Figure 1:**
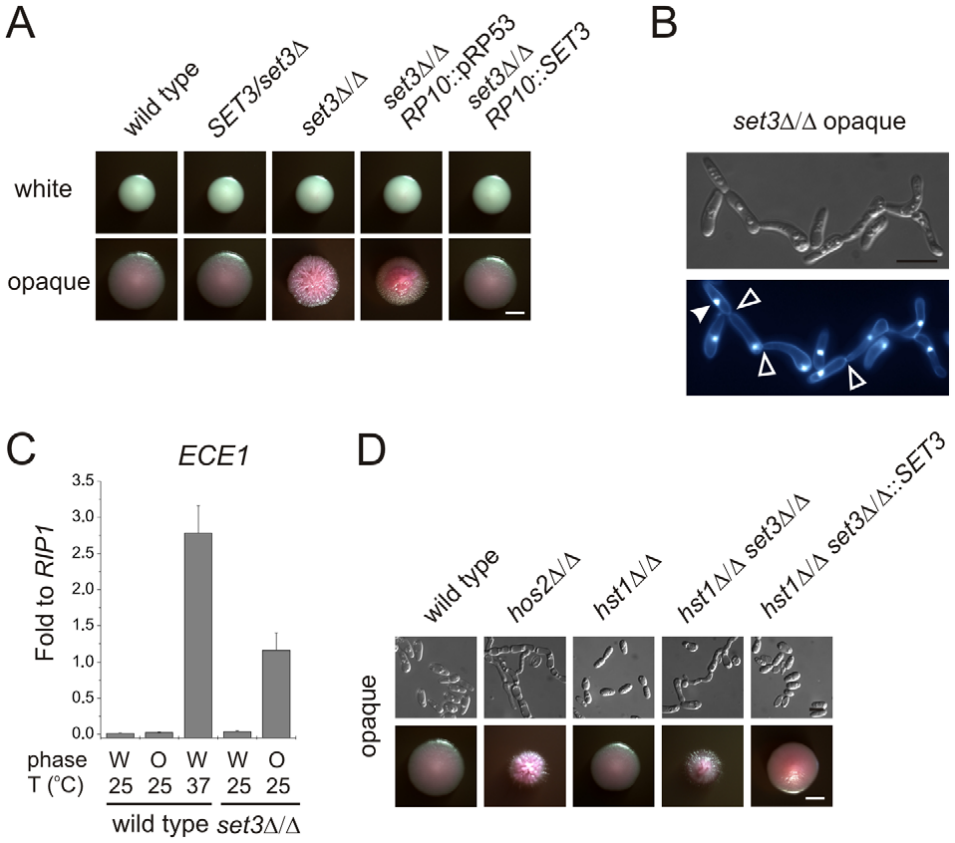
*MTL* homozygous *set3*Δ/Δ cells filament specifically in the opaque phase. (A) Colony morphologies. *MTL* **a**/**a** *set3*Δ/Δ cells grow as wrinkled colonies in the opaque but not in the white phase at 25°C on Lee's agar plates containing 5 µg/ml Phloxin B, staining opaque cells pink. Images were taken after 5 days of incubation. Scale bar corresponds to 2 mm. (B) Opaque phase filaments of *MTL* **a**/**a** *set3*Δ/Δ cells on Lee's medium display pseudohyphal characteristics. Cells are elongated and constrictions are visible where two daughter cells stay attached after cell division. Bold arrowheads indicate nuclei stained with DAPI. Cell wall is stained with Calcofluor White. Empty arrowheads indicate cell wall constrictions. Scale bar corresponds to 5 µm. (C) The *MTL* **a**/**a** *set3*Δ/Δ mutant expresses the filament-specific *ECE1* transcript at high levels in the opaque phase. qRT-PCR analysis was performed with cDNA samples derived from colonies shown in Figure1A. In addition, *ECE1* expression level in white phase *MTL* **a**/**a** wild type cells grown on Lee's medium at 37°C for three days is added as a control. Transcript levels were normalized against the expression level of *RIP1*. qRT-PCR reactions were performed in triplicates and RNA isolated from two independent cultures were analyzed. Data are shown as mean + SD. (D) Colony morphologies of additional mutants of the putative Set3/Hos2 complex. Opaque phase *MTL* **a**/**a** cells deleted for the core subunit *HOS2* (see text) form wrinkled colonies on Lee's medium at 25°C, similar to *set3*Δ/Δ cells, whereas mutants lacking the peripheral subunit *HST1* form smooth colonies. In addition, *SET3* is epistatic to *HST1*. Images were taken after five days of incubation. Scale bar corresponds to 2 mm.

In a previous study, we reported that *SET3* and *HOS2* are key regulators of white-opaque switching in *MTL* homozygous *C. albicans* strains [25]. Surprisingly, *MTL* **a**/**a** *set3*Δ/Δ opaque cells show an additional phenotype: they form wrinkled colonies on Lee's agar plates as opposed to the smooth colonies formed by wild type opaque cells and *SET3*/*set3*Δ heterozygotes (Figure 1A). Reintegration of one copy of *SET3* at the *RP10* locus rescued the phenotype, whereas the integration of a control vector at the *RP10* locus did not (Figure 1A).

Microscopic inspection revealed that wrinkled opaque colonies formed by *set3*Δ/Δ cells consisted of mostly filamentous structures with pseudohyphal characteristics [29]. Namely, the cells were elongated and longer than wild type opaque cells, and constrictions were visible at mother-daughter cell junctions (Figure 1B and S2B). In addition, *set3*Δ/Δ opaque cells expressed the filament-specific gene *ECE1* [30] at high levels, indicating an active filamentation program (Figure 1C).

Given the strong conservation of the yeast Set3C in *C. albicans*, we tested whether hyperfilamentation occurs upon deletion of other subunit-genes of the putative complex. Opaque cells lacking the core subunit Hos2 also gave rise to wrinkled colonies consisting of mostly pseudohyphae (Figure 1D). On the other hand, opaque cells lacking the peripheral subunit Hst1 formed smooth colonies, whereas opaque *set3*Δ/Δ *hst1*Δ/Δ cells displayed wrinkled colonies, which reverted to smooth ones upon complementing the *SET3-*deletion (Figure 1D). Thus, in *C. albicans* the Set3C is likely to be of similar architecture as in *S. cerevisiae*. Importantly, the data indicate that loss of a functional Set3C triggers hyperfilamentation of opaque phase cells.

### Loss of *SET3* promotes filamentation at an elevated temperature

**Figure 2:**
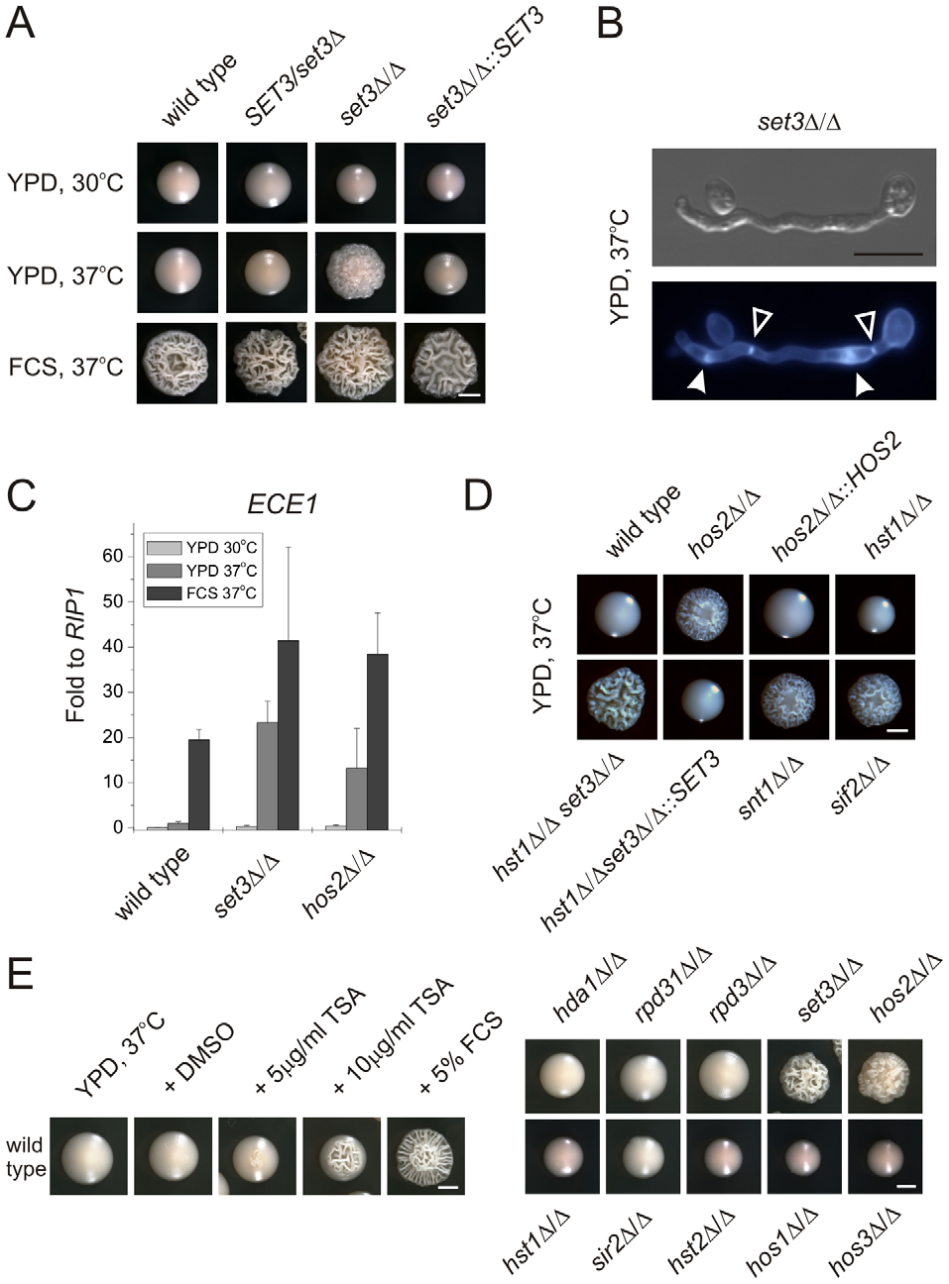
Loss of *SET3* promotes filamentation in *C. albicans*. (A) Colony morphologies. *set3*Δ/Δ cells form wrinkled colonies on YPD at 37°C. Strains were grown for three days. FCS stands for YPD supplemented with 10% fetal calf serum. Scale bar corresponds to 2 mm. (B) The filaments of *set3*Δ/Δ cells on YPD at 37°C display true hyphal characteristics: parallel cell walls with perpendicular septa. Bold arrowheads indicate nuclei stained with DAPI. Cell wall is stained with Calcofluor White. Empty arrowheads indicate the septa. Scale bar corresponds to 5 µm. (C) *set3*Δ/Δ and *hos2*Δ/Δ mutants express the filament-specific *ECE1* transcript at high levels on YPD at 37°C. qRT-PCR analysis was performed with cDNA samples derived from the colonies shown in Figure2A. Transcript levels were normalized against the expression level of *RIP1*. qRT-PCR reactions were performed in triplicates and RNA isolated from two independent cultures were analyzed. Data are shown as mean + SD. (D) Colony morphologies of additional mutants of the Set3 Complex. Cells deleted for the core subunits *HOS2, SNT1* and *SIF2* form wrinkled colonies on YPD at 37°C, whereas the deletion cells lacking the peripheral subunit *HST1* form smooth colonies. In addition, *SET3* is epistatic to *HST1*. Images were taken after three days of incubation. Scale bar corresponds to 2 mm. (E) (left panel) Trichostatin A treatment enhances filamentation. The displayed strain is a *MTL* **a**/**a** strain. (right panel) Trichostatin A treatment is phenocopied by genetic disruption of the Set3C, but none of the other putative histone deacetylases. Images were taken after three days of incubation at 37°C. Scale bar corresponds to 2 mm.

*SET3* has been previously identified in a large-scale haploinsufficiency screen as a modulator of filamentation in *MTL* heterozygous *C. albicans* cells [31]. Thus, we constructed several independent hetero- and homozygous *SET3* deletion strains in an *MTL* **a**/α background, and compared the morphologies of wild type and deletion mutants on several standard liquid and solid laboratory media. Most notably, the *set3*Δ/Δ strain displayed hyperfilamentous growth on solid YPD at 37°C (Figure 2A). YPD at 30°C was used as standard medium supporting yeast phase growth, whereas YPD supplemented with 10% fetal calf serum (FCS) were standard conditions that promote filamentation (Figure 2A). *set3*Δ/Δ mutants showed a mild hyperfilamentation phenotype on filament-inducing Lee and Spider plates at 37°C. However, wild type and *set3*Δ/Δ did not show any apparent differences in liquid cultures of all conditions tested (Figure S1). In addition, *set3*Δ/Δ cells were more invasive on some solid media (Figure S1). Notably, a haploinsufficiency phenotype of *SET3*/*set3*Δ cells as reported earlier [31] was not observed in our experimental settings (Figure 1A and 2A), but we observed a mild haploinsufficiency effect at temperatures above 37°C (data not shown).

Microscopic inspection revealed that wrinkled *set3*Δ/Δ colonies on YPD plates at 37°C consisted of mainly filaments characterized by parallel cell walls and perpendicular septa, the hallmarks of true hyphae (Figure 2B and S2A) [29]. Furthermore, under these conditions, filament-specific *ECE1* mRNA levels in *set3*Δ/Δ colonies were upregulated, confirming an active filamentation program (Figure 2C).

Cells lacking the putative core subunits Hos2, Snt1 and Sif2 also gave rise to wrinkled colonies consisting of mostly true hyphae on YPD at 37°C (Figure 2D), and *hos2*Δ/Δ cells expressed *ECE1* at comparable levels as *set3*Δ/Δ cells (Figure 2C). By contrast, *hst1*Δ/Δ cells formed smooth colonies under these conditions. Moreover, the *set3*Δ/Δ *hst1*Δ/Δ double mutant formed wrinkled colonies, which was reverted upon restoring *SET3* (Figure 2D), confirming the notion of a conserved Set3C architecture in *C. albicans*.

As expected, the hyperfilamentation on YPD at 37°C was independent of *MTL*-zygosity. Indeed, *MTL* **a**/**a** wild type and mutant white phase strains showed the same characteristics as the respective *MTL* **a**/α isolates (Figure S2C), supporting the general view that the same genetic mechanisms regulate filamentation in both *MTL* heterozygous and *MTL* homozygous white phase cells.

If loss of Set3C function is the cause of the hyperfilamenting phenotype, biochemical inhibition of the catalytic activity should mimic the loss of the catalytic subunit. Therefore, we examined the morphological development of wild type *C. albicans* strains in the presence of the histone deacetylase inhibitor Trichostatin A (TSA) [32]. Surprisingly, we found that 10 µg/ml TSA supplementation of solid YPD medium strongly induced filamentation at 37°C (Figure 2E). We then examined single deletion *C. albicans* strains lacking all putative histone deacetylase under identical growth conditions in the absence of TSA. Most strikingly, only the loss of the Set3 and Hos2 deacetylase genes induced the yeast-hyphae morphogenetic conversion (Figure 2E). These results demonstrate that inhibition of histone deacetylase activity by TSA is phenocopied by the loss of the Set3 and Hos2 core subunits of the *C. albicans* Set3C but not of any other histone deacetylase. Taken together, these data strongly suggest that lack of a functional Set3C causes a hyperfilamentation phenotype in *set3*Δ/Δ and *hos2*Δ/Δ mutants.

Several transcription factors involved in morphogenesis, including the positive regulator *EFG1*, as well as the negative regulators *NRG1* or *SSN6,* are themselves regulated at the transcriptional level upon serum-induced yeast-to-hypha transition [12], [33], [34]. Therefore, we tested whether repression of *SET3* or *HOS2* is associated with the morphological conversion of wild type cells. However, we found no significant differences between *SET3* and *HOS2* mRNA levels in yeast and hyphal cells (Figure S3). These results confirm that a mechanism other than transcriptional inactivation of *SET3* or *HOS2* must constitute a trigger driving yeast-to-hypha conversion.

### Deletion of *EFG1* suppresses the hyperfilamentation of *set3*Δ/Δ and *hos2*Δ/Δ mutants

**Figure 3:**
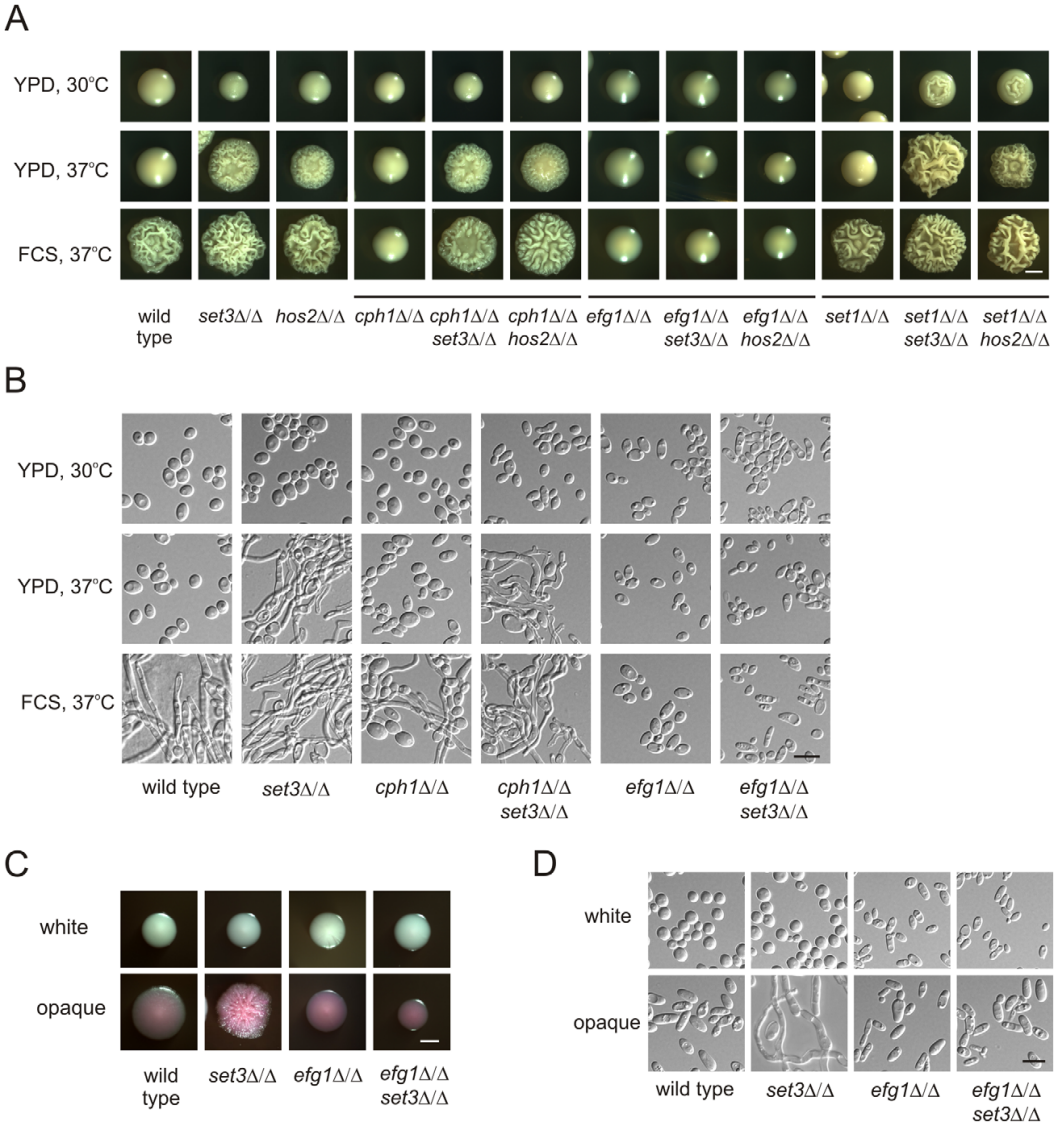
Lack of *EFG1* suppresses hyperfilamentation of *set3*Δ/Δ and *hos2*Δ/Δ mutants. (A) Colony morphologies of mutant strains with the indicated genotypes. Loss of *CPH1* or *EFG1* compromises the ability of cells to form filaments even under serum induction. *EFG1* deletion is epistatic to the deletion of *SET3* or *HOS2*, whereas *CPH1* deletion is hypostatic. Deletion of *SET1* in *set3*Δ/Δ and *hos2*Δ/Δ mutants has a mild synergistic effect. Strains were grown for three days on the media indicated. FCS stands for YPD supplemented with 10% fetal calf serum. Scale bar corresponds to 2 mm. (B) Microscopic analysis of the colonies shown on Figure 3A. On YPD at 37°C the wrinkled colonies of *cph1*Δ/Δ *set3*Δ/Δ cells consists of a mixture of yeast cells and hyphae, while *efg1*Δ/Δ *set3*Δ/Δ show the slightly elongated morphology of *efg1*Δ/Δ cells irrespective of the presence of serum. Scale bar corresponds to 5 µm. (C) Colony morphologies of mutant strains with the indicated genotypes. In opaque phase cells, *EFG1* deletion is epistatic to the loss of *SET3*. Strains were grown at 25°C on Lee's agar plates containing 5 µg/ml Phloxin B. Images were taken after 5 days of incubation. Scale bar corresponds to 2 mm. (D) Microscopic analysis of the colonies shown in Figure 3C. Contrary to opaque *set3*Δ/Δ cells, no filamentous structures are present in the colonies formed by opaque *efg1*Δ/Δ or *efg1*Δ/Δ *set3*Δ/Δ cells. Scale bar corresponds to 5 µm.

Does the Set3C represent an independent pathway regulating morphogenesis or is it a component of a previously characterized signaling pathway? To investigate the epistatic relationships between *SET3*, *HOS2* and the previously characterized positive regulators *CPH1* and *EFG1* [8], [9], we analyzed the phenotypes of relevant double deletion mutants. The *cph1*Δ/Δ *set3*Δ/Δ and *cph1*Δ/Δ *hos2*Δ/Δ double mutants formed hyphae on YPD plates at 37°C, as well as in response to serum. Thus, the combined loss of *CPH1* and *SET3* or *CPH1* and *HOS2* resembled the single deletion phenotypes of *set3*Δ/Δ and *hos2*Δ/Δ cells (Figure 3A and B). On the other hand, the *efg1*Δ/Δ *set3*Δ/Δ and *efg1*Δ/Δ *hos2*Δ/Δ double mutants failed to filament, even upon induction with serum; therefore, loss of *EFG1* was epistatic to the deletion of *SET3* or *HOS2* (Figure 3A and B).

In addition, we analyzed the genetic relationship between *SET3*, *HOS2* and *SET1*. *C. albicans SET1* is a histone methyltransferase gene [35], acting in the same pathway as *SET3* and *HOS2* regulating the frequency of white opaque switching [25]. Whereas in white-opaque switching regulation, *SET1* is epistatic to *SET3* and *HOS2* [25], we noticed a slight enhancement in filament formation in *set1*Δ/Δ *set3*Δ/Δ and *set1*Δ/Δ *hos2*Δ/Δ double mutants (Figure 3A). Furthermore, loss of *EFG1* was epistatic to the loss of *SET3* in opaque phase cells, as well (Figure 3C and 3D). Taken together, these results suggest that *SET3* and *HOS2* are acting in a different pathway than *CPH1* or *SET1.* Nevertheless, Set3C may interfere with Efg1-dependent signaling, resulting in hyperfilamentatation phenotypes of *set3*Δ/Δ and *hos2*Δ/Δ single mutants.

### Loss of *SET3* or *HOS2* enhances the induction of *EFG1*-dependent target genes

The epistasis relations excluded the involvement of a Cph1-dependent mechanism contributing to the effect of *SET3* and *HOS2* deletions, but *EFG1* remained as a possibility. In addition, we also wanted to investigate whether the lack of *SET3* or *HOS2* interferes with the function of Tup1, a known transcriptional repressor promoting yeast-phase growth in *C. albicans* [11]. Notably, Tup1 in *S. cerevisiae* was shown to directly interact with Hos2, and loss of Hos2 was shown to compromise Tup1-dependent gene repression [36].

**Figure 4:**
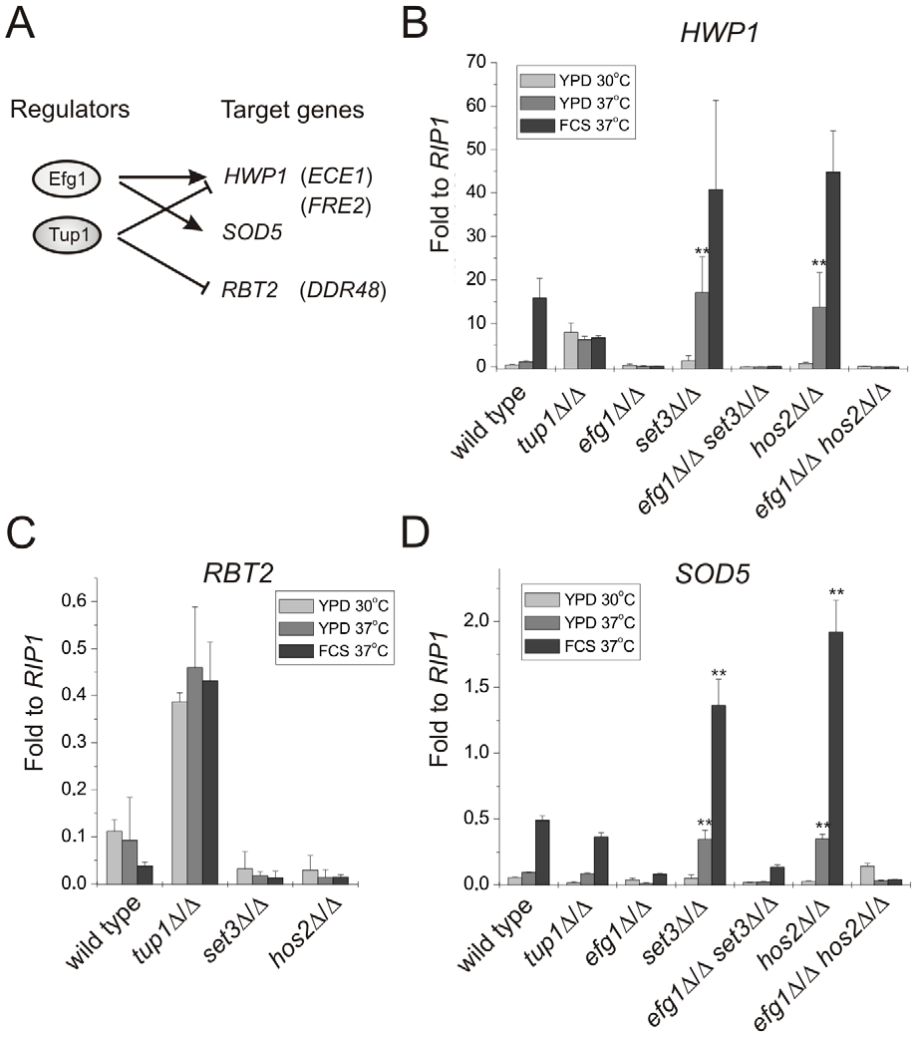
Loss of *SET3* or *HOS2* enhances induction of *EFG1*-dependent target genes. (A) Scheme of the experimental approach. Expression profiles of the genes in parentheses are shown on Figure S4. qRT-PCR analysis was performed with cDNA samples derived from the colonies shown in Figure 2A and 3A. Transcript levels were normalized against the expression level of *RIP1*. qRT-PCR reactions were performed in triplicates and RNA isolated from two independent cultures were analyzed. Data are shown as mean + SD. (B) The expression of *HWP1* is strongly induced in *set3*Δ/Δ and *hos2*Δ/Δ cells even on YPD at 37°C. *HWP1* expression is abolished once *EFG1* is deleted both in wild type and *set3*Δ/Δ or *hos2*Δ/Δ cells. Double asterisk indicates statistical significance of P<0.01 relative to wild type cells cultured under identical conditions (Student's t-test). (C) *RBT2* is repressed by Tup1, but not by Set3 or Hos2 under all conditions tested. (D) The expression of *SOD5* is strongly induced in *set3*Δ/Δ and *hos2*Δ/Δ cells upon a mild (YPD, 37°C) or strong (FCS, 37°C) inductive stimulus. Elevated *SOD5* expression requires *EFG1*. Double asterisk indicates statistical significance of P<0.01 relative to wild type cells cultured under identical conditions (Student's t-test).

Therefore, we chose several marker genes whose expression is regulated by Efg1, Tup1 or both of them, and analyzed their mRNA levels in *set3*Δ/Δ and *hos2*Δ/Δ mutants (Figure 4A) using qRT-PCR. *HWP1* is a filament-specific gene strongly induced by serum [37] or lack of *TUP1* [38]. In addition, serum induction of *HWP1* is strictly *EFG1*-dependent (Figure 4B) [37]. In the absence of *SET3* or *HOS2,* cells express high *HWP1* levels even on YPD at 37°C, with about 15-fold differences between wild type and *set3*Δ/Δ or *hos2*Δ/Δ cells (P<0.01), indicating an active hyphal program. Moreover, elevated *HWP1*-expression is completely abolished once *EFG1* is also deleted in *set3*Δ/Δ or *hos2*Δ/Δ cells (Figure 4B). In addition, qualitatively identical results were obtained using additional hyphae-specific markers such as *ECE1* and *FRE2* (Figure S4).

*RBT2* is a gene repressed by Tup1. However, *RBT2* expression does not change upon serum induction, excluding an Efg1-mediated control of *RBT2* (Figure 4C) [39]. In the absence of *SET3* or *HOS2,* cells express low levels of *RBT2* under all conditions tested, comparable to wild type cells, indicating that their absence does not interfere with Tup1-mediated repression of *RBT2*. Moreover, *DDR48* mRNA levels also show a qualitatively identical expression pattern in these mutants (Figure S4).

Finally, *SOD5* is expressed at low levels in yeast phase but is induced in an *EFG1*-dependent manner in serum-induced hyphae, and induction is maintained in *tup1*Δ/Δ cells (Figure 4D) [40]. Interestingly, we found that *set3*Δ/Δ and *hos2*Δ/Δ cells express *SOD5* at levels higher than wild type cells on YPD at 37°C (about 3.5-fold relative to wild type, P<0.01) or in the presence of serum at 37°C (about 3.3-fold relative to wild type, P<0.01) (Figure 4D). Most importantly, the elevated *SOD5* mRNA levels, both in *set3*Δ/Δ and *hos2*Δ/Δ cells, required *EFG1* (Figure 4D). Taken together, these results demonstrate that loss of *SET3* or *HOS2* strongly enhances the expression of *EFG1*-dependent genes. In other words, genes repressed by Efg1 in yeast phase cells are induced upon loss of *SET3* or *HOS2*, with *TUP1*-dependent targets remaining unaffected.

### Hyperfilamentation of *set3*Δ/Δ cells is suppressed by adenine supplementation

**Figure 5:**
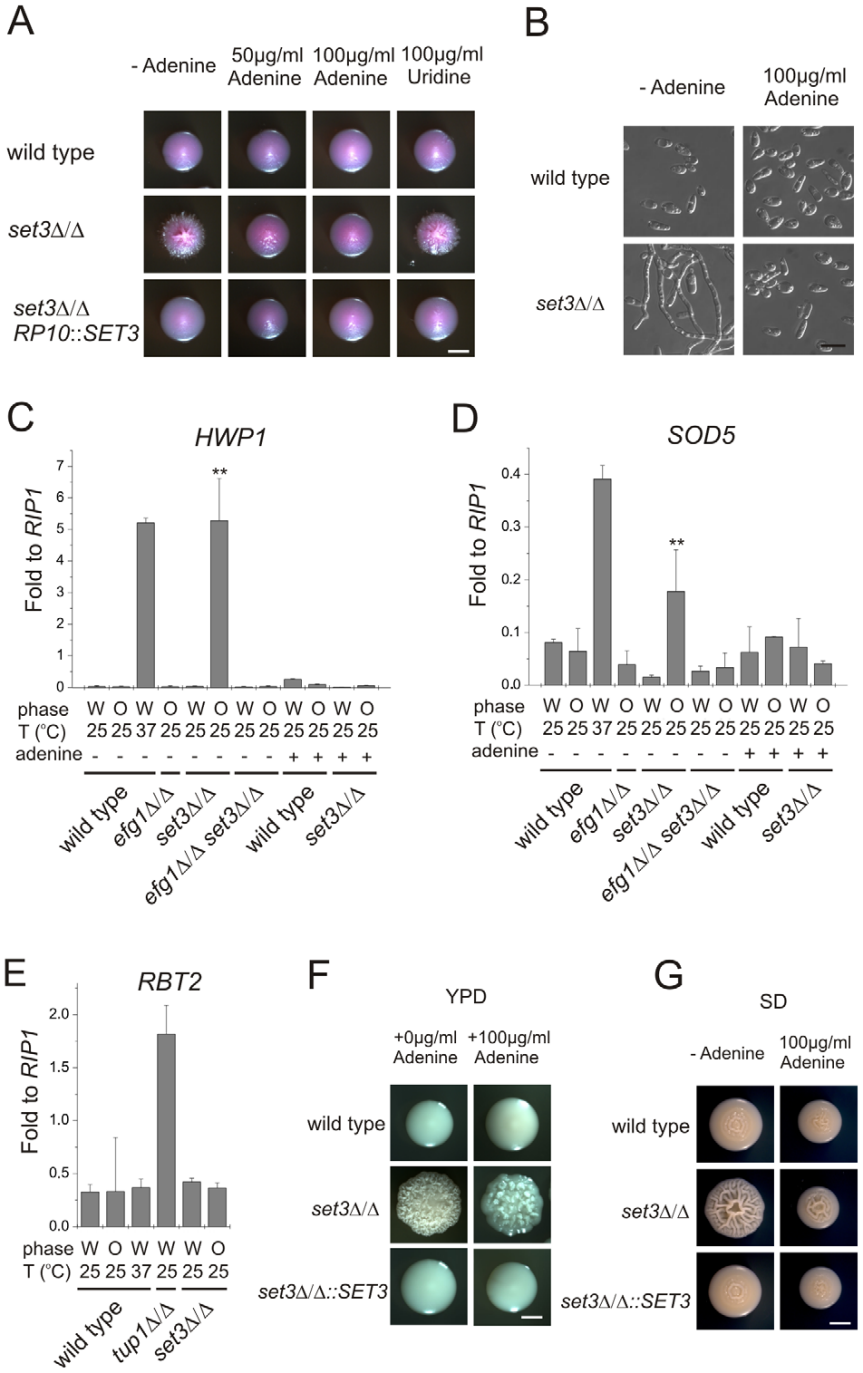
Adenine supplementation suppresses hyperfilamentation of *set3*Δ/Δ mutants. (A) Colony morphologies of opaque phase *MTL* **a**/**a** strains. Opaque *set3*Δ/Δ cells form smooth colonies on Lee's medium supplemented with adenine. Images were taken after five days of incubation at 25°C on Lee's agar plates containing 5 µg/ml Phloxin B. (B) Microscopy of the colonies shown in Figure 5A. The opaque *MTL* **a**/**a** *set3*Δ/Δ cells do not filament in the presence of 100 µg/ml adenine. Scale bar corresponds to 5 µm. In panels (C), (D) and (E), the logic for the expression analysis is described in Figure 4A. qRT-PCR analysis was performed with cDNA samples derived from the colonies shown in Figure 3C and Figure 5A. Transcript levels were normalized against the expression level of *RIP1*. qRT-PCR reactions were performed in triplicates and RNA isolated from two independent cultures were analyzed. Data are shown as mean + SD. (C) *HWP1* expression is strongly induced in opaque phase *set3*Δ/Δ cells, but the induction is suppressed by deletion of *EFG1* or by supplementing the medium with 100 µg/ml adenine. Double asterisk indicates statistical significance of P<0.01 relative to wild type cells of the same phase cultured under identical conditions (Student's t-test). (D) Expression of *SOD5* is induced in *set3*Δ/Δ opaque cells, but the induction is suppressed by deletion of *EFG1* or by supplementing the medium with 100 µg/ml adenine. Double asterisk indicates statistical significance of P<0.01 relative to wild cells of the same phase cultured under identical conditions (Student's t-test). (E) *RBT2* is repressed by Tup1, but not by Set3 or Hos2. (F) Colony morphologies on YPD medium without or with 100 µg/ml adenine added. Scale bar corresponds to 2 mm. (G) Colony morphologies on SD medium without or with 100 µg/ml adenine added. Scale bar corresponds to 2 mm.

In a previous study, we have shown that adenine is an environmental factor that modulates the frequency of the opaque to white transition *in vitro* requiring *SET3* [25]. We also made the striking observation that opaque isolates of the *MTL* **a**/**a** *set3*Δ/Δ cells did not form pseudohyphae on adenine-supplemented Lee's medium at 25°C (Figure 5A and B). The effect of adenine was both dose-dependent and specific; 100 µg/ml adenine completely reverted cells to yeast phase growth, while the same concentration of uridine failed to do so (Figure 5A and B).

To analyze the effect of adenine supplementation at the gene expression level, we first confirmed the induction of the *EFG1*-dependent targets in opaque *MTL* **a**/**a** *set3*Δ/Δ mutants. As expected, *HWP1* was strongly induced in *set3*Δ/Δ opaque but not in white cells (around 200-fold relative to wild type, P<0.01); nevertheless, *HWP1* expression was reduced back to wild type levels by deleting *EFG1* or upon addition of adenine to the medium (Figure 5C). In addition, *ECE1* levels showed a qualitatively similar pattern (Figure S4). Furthermore, *SOD5* was also induced in *set3*Δ/Δ opaque cells (around 2.6-fold relative to wild type, P<0.01), and its induced expression was also reduced in *efg1*Δ/Δ *set3*Δ/Δ cells or *set3*Δ/Δ cells grown in the presence of adenine (Figure 5D). The expression level of the Tup1-target *RBT2* was again unaffected (Figure 5E).

Is the hyperfilamentation phenotype of white *set3*Δ/Δ cells on YPD at 37°C also reverted by adenine supplementation? Unfortunately, it is not possible to address this question directly, because the yeast extract present in YPD contains adenine [41]. Hence, as expected, additional 100 µg/ml adenine supplementation on YPD had no effect on the phenotype of *set3*Δ/Δ cells at 37°C (Figure 5F). However, if the strains were cultured on SD medium, which has defined components and is devoid of adenine yet supports yeast-phase growth of wild type cells, we observed hyperfilamentation of *MTL* **a**/α *set3*Δ/Δ cells at 37°C. This was reverted by adding 100 µg/ml adenine to SD or complementation of one allele of *SET3* (Figure 5G). Taken together, these results demonstrate that both the opaque and white filamentation phenotypes of *set3*Δ/Δ cells can be suppressed by exogenous adenine provided in the growth medium.

### The *set3*Δ/Δ cells have a hyperactive cAMP/PKA pathway

**Figure 6:**
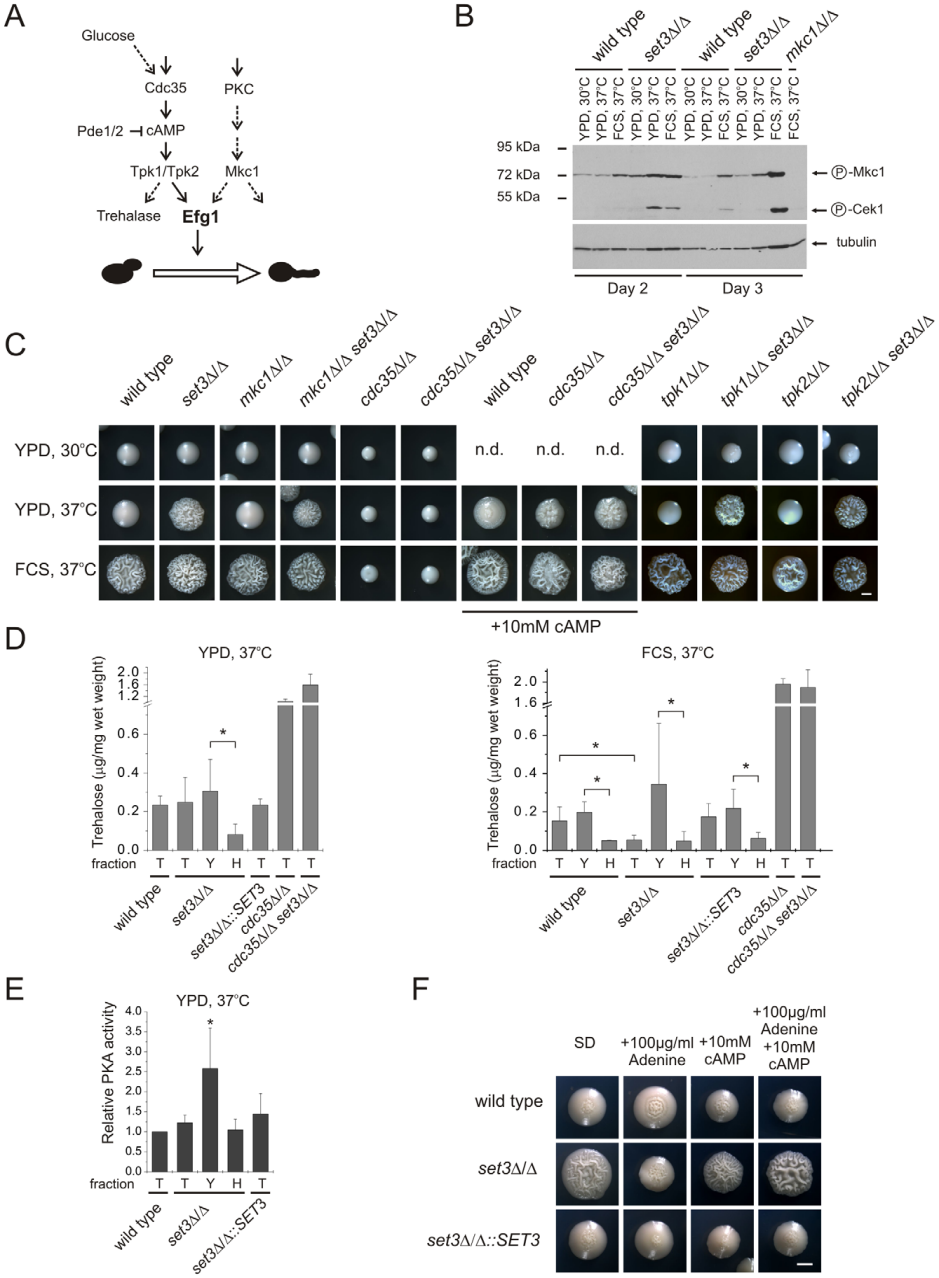
The *set3*Δ/Δ and *hos2*Δ/Δ cells have a hyperactive cAMP/PKA pathway. (A) Simplified scheme of signaling pathways converging at Efg1. Dashed lines indicate implied or indirect connections. (B) Western blot analysis of phosphorylated MAP kinases. Deletion of *SET3* is associated with increased level of phosphorylated Mkc1, indicating active PKC signaling (compare lanes 8 and 11). The antibody also recognizes phosphorylated Cek1, the upstream MAP kinase of Cph1 [67]. (C) Colony morphologies of mutant strains with the indicated genotypes. *SET3* is epistatic to *MKC1, TPK1* and *TPK2* but hypostatic to *CDC35*. Images were taken after three days of incubation except for the *cdc35*Δ/Δ and *cdc35*Δ/Δ *set3*Δ/Δ strains, which were incubated for four days. FCS stands for YPD supplemented with 10% fetal calf serum. Scale bar corresponds to 2 mm. (D) Trehalose content of colonies grown on YPD at 37°C (left panel). Although the total colonies have similar trehalose levels, the hyphal fraction of the *set3*Δ/Δ cells contains about 4-times less trehalose as the yeast fraction, indicating a history of elevated PKA activity. When grown on plates supplemented with FCS at 37°C (right panel), *set3*Δ/Δ colonies contain about 3-times less trehalose than wild type or *set3*Δ/Δ::*SET3* colonies. Moreover, all filamentous fractions contain about 4-times less trehalose than the corresponding yeast fractions, indicating a history of elevated PKA activity. “T”: total, “Y”: yeast, “H”: hyphal fraction. Data are displayed as mean + SD of three independent experiments. Asterisk indicates statistical significance of P<0.05 (Student's t-test). (E) Protein kinase A activities of cell extracts derived from the indicated colonies. Data are normalized against the activity level of wild type extracts, and are displayed as mean + SD of three independent experiments. “T”: total, “Y”: yeast, “H”: hyphal fraction. Asterisk indicates statistical significance of P<0.05 (Student's t-test). (F) Colony morphologies. Exogenous cAMP rescues the sensitized morphogenetic potential of *set3*Δ/Δ cells in the presence of adenine. Cultures were grown for 5 days at 37°C on SD medium. Scale bar corresponds to 2 mm.

Why are the Efg1-dependent target genes activated upon disruption of the Set3C? Efg1 receives input information from two signaling cascades. First, Efg1 is a downstream target of the cAMP/protein kinase A (PKA) signaling pathway. Briefly, this pathway transmits nutritional signals and involves the activation of the adenylyl-cyclase Cdc35 initiating cAMP synthesis, thereby activating PKA. *C. albicans* harbors two functional PKA catalytic subunits, Tpk1 and Tpk2 [42]. Second, Mkc1, the central MAP kinase of the protein kinase C (PKC) cell integrity pathway sensing cell wall damage, is also proposed to regulate Efg1-dependent morphogenesis [43]. Consequently, we tested the contribution of PKA and PKC signaling to the hyperfilamenting phenotypes of *set3*Δ/Δ (Figure 6A).

To assess activation of the PKC pathway, we performed Western blot analysis of the active, phosphorylated form of Mkc1. As expected, under filament-inducing conditions, both wild type and *set3*Δ/Δ cells harbored activated and thus phosphorylated Mkc1 (Figure 6B). Surprisingly, a high level of phosphorylated Mkc1 was also apparent in the *set3*Δ/Δ colonies after three days of incubation on YPD at 37°C, indicating a hyperactive PKC pathway (Figure 6B). However, we subsequently found that a lack of *SET3* was epistatic to the deletion of *MKC1*, since the *mkc1*Δ/Δ *set3*Δ/Δ double mutant displayed filamentous growth on solid YPD at 37°C similar to the *set3*Δ/Δ mutant (Figure 6C). These data demonstrate that although the PKC pathway appears active in *set3*Δ/Δ cells on YPD at 37°C, it is not the cause, but rather a consequence of the active filamentation program.

To address whether an active cAMP/PKA pathway is required for the hyperfilamenting phenotype of *set3*Δ/Δ cells, we performed epistasis experiments of *SET3* with *CDC35* encoding the adenylyl-cyclase, as well as *TPK1* and *TPK2* encoding the two PKA catalytic subunits. As shown on Figure 6C, lack of *CDC35* prevented both serum- and temperature-induced filamentation of wild type and *set3*Δ/Δ cells which was reverted upon supplementing the media with 10 mM cAMP. These data demonstrates that hyperfilamentation of *set3*Δ/Δ cells requires a functional cAMP/PKA pathway. On the other hand, deletion of *SET3* was epistatic to the deletion of either *TPK1* or *TPK2*, yet these results can be explained by the functional redundancy of the two PKA genes.

To link hyperfilamentation in the absence of Set3C with an increased activity of the cAMP/PKA pathway, we measured the trehalose content of wild type and mutant cultures. Trehalose is a disaccharide present in many yeast species; it is degraded by the neutral trehalase enzyme, which is activated by PKA [44], [45]. As expected, we did not observe differences in the trehalose contents of wild type, *set3*Δ/Δ, *set3*Δ/Δ::*SET3* and *hos2*Δ/Δ cells after growth for two and three days on solid YPD at 30°C supporting yeast phase growth (Figure S5A). Surprisingly, we failed to detect any differences between the four strains on solid YPD at 37°C, where *set3*Δ/Δ and *hos2*Δ/Δ cells readily filament (Figure 6D, S5A). However, *set3*Δ/Δ cells contained around 3-fold less (P<0.05) trehalose than wild type or *set3*Δ/Δ::*SET3* heterozygous colonies when grown for three days on filament-inducing, FCS-supplemented plates (Figure 6D, S5A). Therefore, we concluded that differences in trehalose levels between yeast cells and filamentous forms may not be detectable because the colonies contain a mixture of both (Figure S2A). Consequently, we developed a filtration-based method to separate the yeast-phase and filamentous cells of colonies, to yeast and hyphal fractions. Strikingly, we found about 4-fold more trehalose in the yeast fraction when compared to the hyphae fraction (P<0.05, Figure 6D) derived from the same colonies of wild type, *set3*Δ/Δ and *set3*Δ/Δ::*SET3* cells when grown on filament-inducing FCS medium at 37°C. Hence, a decreased trehalose content may reflect a history of PKA-activation, once the yeast-filament conversion has been initiated. In good agreement with this suggestion, a similar decrease in trehalose content was also detectable between the yeast and hyphal fractions of *set3*Δ/Δ cells grown on YPD medium at 37°C (P<0.05, Figure 6D).

To directly assess whether at some stage of differentiation an elevated PKA activity can be detected in the hyperfilamentous *set3*Δ/Δ cells, we performed PKA activity assays using total cell extracts derived from wild type, *set3*Δ/Δ and *set3*Δ/Δ::*SET3* cells grown on YPD at 37°C. In these experiments, a fluorescently labeled peptide is phosphorylated by PKA, followed by electrophoretic separation of phosphorylated and non-phosphorylated peptides and quantification by spectrophotometry (see Figure S5B and details in Materials and Methods). Surprisingly, we detected about 2-fold higher PKA activity of the yeast fraction of *set3*Δ/Δ cells when compared to the wild type (P<0.05, Figure 6E), indicating that this fraction contains a subset of cells harboring an elevated level of active PKA, although this difference is undetectable when comparing whole colonies (Figure 6E). Taken together, these results demonstrate that the hyperfilamentous phenotype of *set3*Δ/Δ cells requires functional cAMP/PKA signaling; differentiated hyphae are associated with a decrease in trehalose, while a subset of *set3*Δ/Δ cells harbor an elevated level of active PKA when compared to wild type cells.

Finally, we tested if suppression of hyperfilamentation in *set3*Δ/Δ cells by exogenous adenine can be rescued by stimulating the cAMP/PKA pathway with exogenous cAMP. Surprisingly, *set3*Δ/Δ cells indeed displayed a hypersensitive morphogenetic potential in the presence of both exogenous adenine and cAMP (Figure 6F), indicating that both adenine and the Set3C can modulate cAMP/PKA signaling, albeit through antagonistic mechanisms.

### Non-fermentable carbon sources revert hyperfilamentation of *set3*Δ/Δ cells

**Figure 7:**
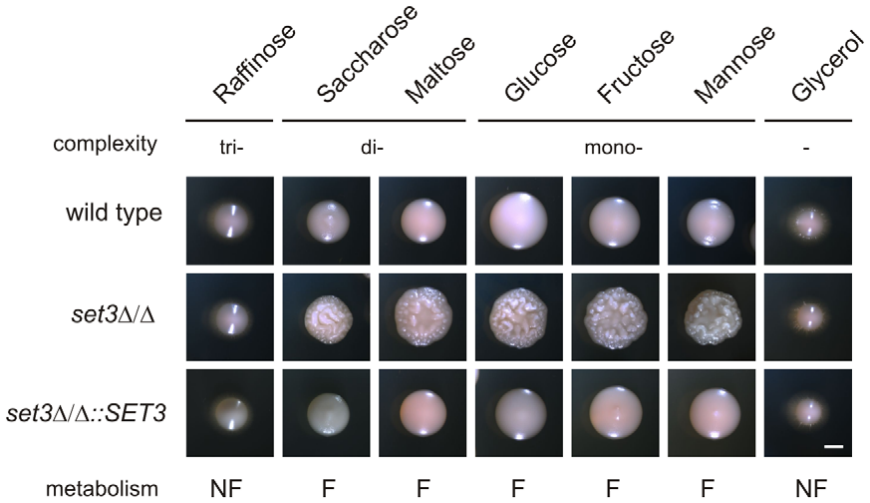
Hyperfilamentation of *set3*Δ/Δ is reverted by non-fermentable carbon sources. Colony morphologies on YP medium supplemented with 2% of the indicated carbon sources. *set3*Δ/Δ display wild type morphology on media containing non-fermentable carbon sources. Since *Candida spp.* do not have a β-lactamase, the cells fail to convert raffinose into fermentable monosaccharides. “F:” fermentable, “NF”: non-fermentable. Cultures were grown for 3 days at 37°C. Scale bar corresponds to 2 mm.

In *S. cerevisiae*, a known substrate of the cAMP/PKA pathway is glucose. Cells cultured on fermentable carbon sources (that can be degraded through glycolysis) are associated with a higher activity of cAMP/PKA signaling, while cells grown on non-fermentable carbon sources are characterized with a lower activity of cAMP/PKA signaling and activation of gluconeogenesis [44] (Figure 6A). Consequently, we tested if limiting cAMP/PKA signaling by non-fermentable carbon sources reverts the hyperfilamentation of *set3*Δ/Δ cells. Indeed, the hyperfilamentation effect was only observed if cells were grown on saccharides whose enzymatic degradation yields glucose. By contrast, *set3*Δ/Δ cells reverted to wild type morphologies in the presence of the non-fermentable carbon sources such as glycerol (Figure 7). A similar suppression was observed when the cells were grown on raffinose as a carbon source (Figure 7). Since yeasts, including *Candida* species do not have a β-lactamase enzyme, they fail to degrade raffinose (consisting of a glucose, galactose and fructose molecule) to fermentable monosaccharides. These results demonstrate that metabolic conditions normally associated with low cAMP/PKA signaling are sufficient to revert the hyperfilamentation effect of the *SET3*-deletion.

### *set3*Δ/Δ cells have a hypersensitive cAMP/PKA signaling pathway

**Figure 8:**
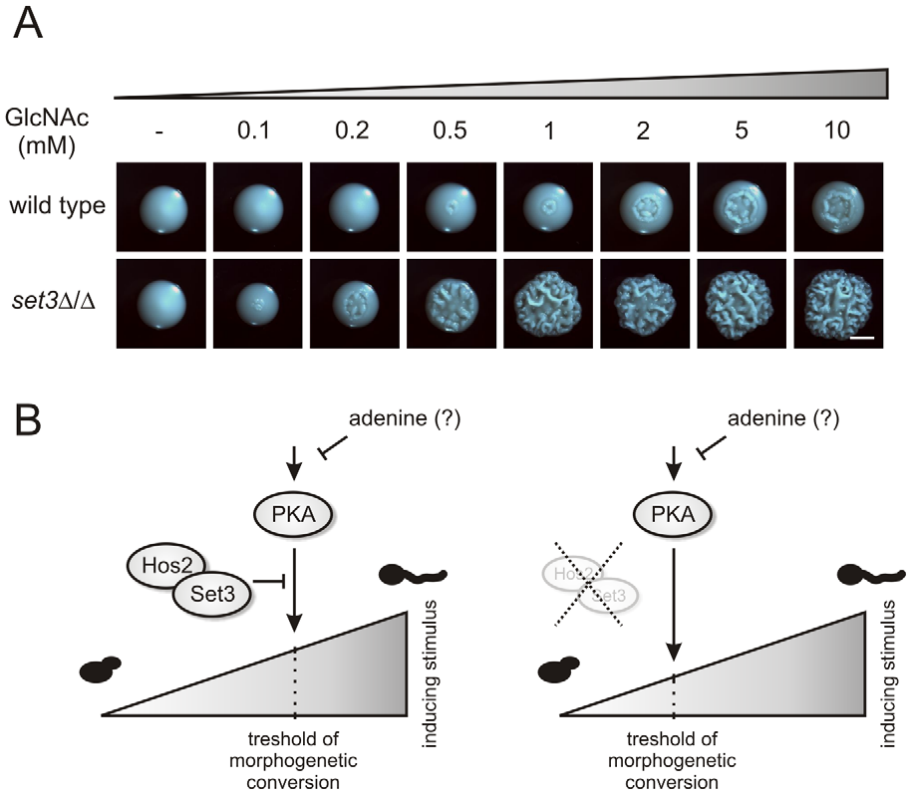
*set3*Δ/Δ cells are a hyperreactive to cAMP/PKA induction by GlcNAc. (A) Colony morphologies of wild type and *set3*Δ/Δ cells grown on YPD at 30°C for 3 days in the presence of the indicated amounts of N-acetylglucosamine (GlcNAc). Scale bar corresponds to 2 mm. (B) The “threshold shift” model for Set3C function in triggering morphogenesis. The cAMP/PKA signaling pathway transmits environmental information, thereby shaping the morphogenetic change. In wild type cells (left panel), the sensitivity of the pathway to adequate signals is antagonized by the SetC. If the Set3C is disrupted or impaired (right panel), the threshold for morphogenetic conversion is shifted, and the pathway responds to milder inducing stimuli by triggering filamentation. In addition, metabolites such as adenine also modulates the activity of the pathway through as yet undisclosed mechanisms.

N-acetylglucosamine (GlcNAc) can activate the cAMP/PKA pathway, and thus is a potent inducer of the yeast-filament transition [5], [46]. To test whether activation of the cAMP/PKA pathway in the hyperfilamentous *set3*Δ/Δ cells result from a pathway being hypersensitive to filament-inducing stimuli, we analyzed the morphological development of wild type and *set3*Δ/Δ cells on solid media containing a gradient of GlcNAc. At 30°C, wild type cells required high amounts (5–10 mM) of GlcNAc to trigger filamentous growth (Figure 8A). By contrast, *set3*Δ/Δ cells displayed massive filamentation already at much lower (0.5–1 mM) concentrations, demonstrating that the cAMP/PKA pathway in this mutant is hypersensitive to inducing stimuli when compared to wild type cells (Figure 8A).

### The *set3*Δ/Δ mutant shows attenuated virulence in a murine infection model

**Figure 9:**
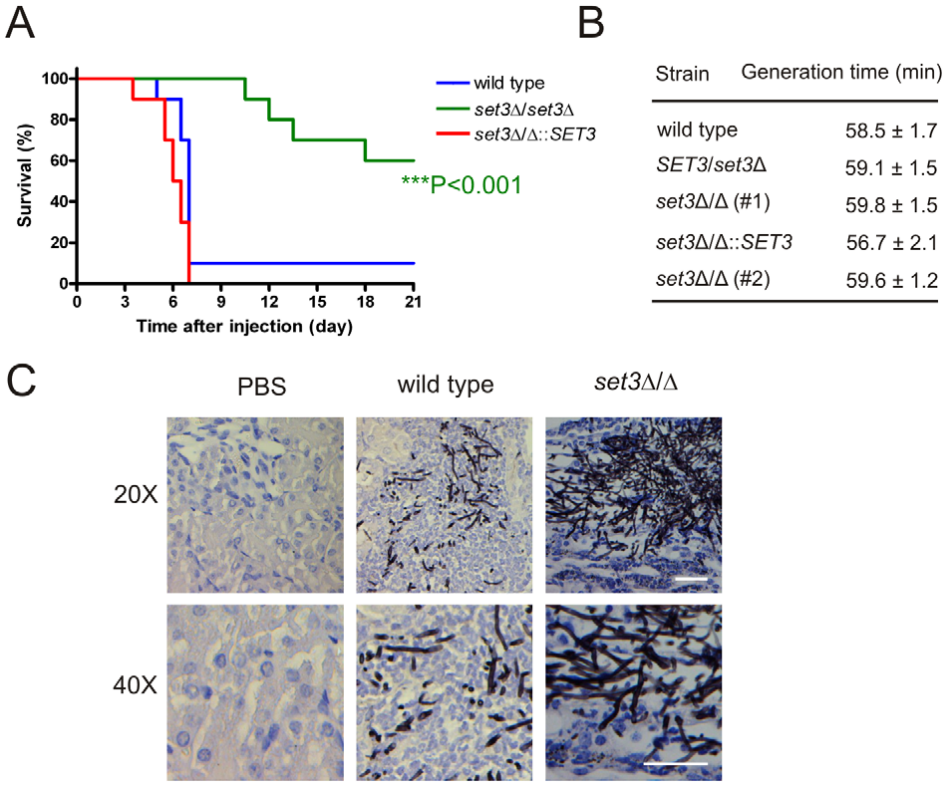
The *set3*Δ/Δ mutant shows attenuated virulence in a murine infection model. (A) Kaplan-Meier survival curves of mice receiving tail vein injections of *MTL* **a**/α wild type, *set3*Δ/Δ and *set3*Δ/Δ::*SET3 C. albicans* strains. Ten mice per *C. albicans* genotype were injected; survival was monitored over three weeks. Statistical significance was determined using the Log-rank test. (B) *set3*Δ/Δ strains do not have a growth defect *in vitro*. Generation times of wild type and *set3*Δ/Δ cells were measured in YPD medium at 30°C as described in Materials and Methods. (C) Histopathology of the cortical part of kidneys of mice infected with wild type or *set3*Δ/Δ *C. albicans* strains on day one after infection. The *set3*Δ/Δ displays hyperfilamentous growth. Tissues were stained with Grocott staining to visualize fungal cells. Counterstaining was performed with Hematoxilin.

The above results demonstrate that the Set3C moderates cAMP/PKA signaling, thereby promoting yeast phase growth. To address whether Set3C-dependent restriction of filamentation is important for virulence of *C. albicans in vivo*, we tested the *set3*Δ/Δ mutant in a mouse model of systemic infection. Consequently, wild type, *set3*Δ/Δ and complemented *set3* strains were injected into the tail vein of 6–8 week old male BALB/c mice. As shown on Figure 9A, 9 out of 10 mice infected with the wild type strain and 10 out of 10 mice infected with the complemented *set3* strain died until day seven after injection. By contrast, mice injected with the *set3*Δ/Δ strain started succumbing to the infection only on day 11, and 6 out of 10 mice were still alive after three weeks (P<0.001, Log rank test). We reproduced the infection experiment once more with another group of 10 mice per *C. albicans* genotype essentially yielding identical results (data not shown). The results unequivocally demonstrate a strongly attenuated virulence of *set3*Δ/Δ cells. To confirm that the virulence defect of *set3*Δ/Δ cells is not caused by a reduced growth rate, we measured the generation times of wild type, heterozygous and homozygous *set3* mutants *in vitro*, but found no significant differences between any of the strains (Figure 9B).

Next, we performed histopathology experiments to address whether the virulence defect of *set3*Δ/Δ mutants is associated with altered morphological development or unusual tissue invasion *in vivo.* In the kidneys of mice infected with wild type *C. albicans*, fungal cells were present as a mixture of both unicellular and filamentous forms on day one after infection. By contrast, *set3*Δ/Δ cells were displaying hyperfilamentous morphologies (Figure 9C). These data demonstrate that *set3*Δ/Δ mutants, despite hyperfilamenting *in vivo*, display attenuated virulence in a murine infection model. These data suggest that the virulence defect is not caused by slower growth but rather by the interference with the morphogenetic conversion.

## Discussion

### The Set3C is a cAMP/PKA-antagonistic repressor of filamentation

Here, we identify the Set3C, an evolutionary conserved histone deacetylase complex, as a repressor of the cAMP/PKA pathway regulating the yeast-to-hypha conversion in *Candida albicans*. We provide several lines of evidence that the hyperfilamentation resulting from removing the Set3C is linked to interference with the cAMP/PKA signaling pathway. First, deletion of the pathway genes *CDC35* and *EFG1* is epistatic to the deletion of *SET3* or *HOS2*. Although, *SET3* appears epistatic to both PKA genes *TPK1* and *TPK2,* it can be explained by the functional redundancy of the Tpk1 and Tpk2 enzymes [47]. Second, we found that differentiated hyphae contain around 4-times less trehalose than yeast-phase cells. Thus, trehalose content is a possible readout to detect the history of PKA activation during the yeast-to-hypha conversion of *C. albicans*. Seemingly contradicting this suggestion, an elevated PKA activity level was detected in the yeast phase fraction of filamentous wrinkled *set3*Δ/Δ colonies; however, this discrepancy appears logical because of the shortcomings of the filtering procedure used for the separation of morphologies. Namely, in filamentous colonies the differentiated hyphae stick together, yet short filaments are still passing through the filter pores and are found in the yeast fraction (data not shown). We believe that during growth on solid surfaces, a subset of cells reaches a yet undefined age or metabolic state, and then commit to the yeast-to-hypha conversion through activation of the cAMP/PKA pathway. This subset of cells that are “poised” to initiate germ tube formation is enriched in the yeast fraction of the filamentous colonies, thus enabling detection of subtle differences in PKA activity. Third, growth on non-fermentable carbon sources, which is associated with low cAMP/PKA signaling, is sufficient to revert the hyperfilamentation effect of the *SET3*-deletion.

In this study, we analyzed two distinct filamentation phenotypes of *set3*Δ/Δ cells to elucidate the mechanism of action of the Set3C, and we propose the following model, which we refer to as the “threshold shift model” (Figure 8B). In wild type cells, the cAMP/PKA pathway and its target transcription factor Efg1 regulate morphogenesis in response to several environmental stimuli [9], [10]. Naturally, molecules triggering morphogenetic conversions do not show a homogenous concentration *in vivo*; therefore, the regulatory pathway must have a certain *sensitivity* to input signals, which in the case of the cAMP/PKA pathway, is determined by (at least) two factors: the concentration of key regulatory components inside the cell and a Set3C-dependent attenuation mechanism. For instance, *EFG1* is rapidly downregulated upon yeast-to-hyphae conversion by serum and elevated temperature [10]. Efg1 binds to its own promoter, and in the absence of Efg1, the endogenous *EFG1* promoter is activated [34]. This auto-inhibitory loop appears crucial for a stable commitment to hyphal growth, because ectopic overexpression of *EFG1* causes hyphal cells to revert to yeast growth by lateral budding [34]. At the same time, the results presented in this study suggest that in the absence of a functional Set3C, the cAMP/PKA pathway responds to milder stimuli to initiate conversion to the filamentous growth (Figure 8A and B).

In our belief, this simple “threshold shift” model is coherent with both phenotypes of the *set3*Δ/Δ mutant for the following reasons. First, white phase *set3*Δ/Δ cells grow predominantly as hyphae on YPD at 37°C as opposed to the wild type cells growing as yeasts (Figure 2, S2A). In this system, YPD at 30°C, YPD 37°C and YPD+serum at 37°C in this order represent three conditions on the horizontal axis of the model analogous to a stimulus-gradient (Figure 8B). Wild type cells, whose cAMP/PKA signaling has wild type *sensitivity,* perceive the YPD at 37°C as being below the threshold required for hyphal conversion. By contrast, *set3*Δ/Δ mutants perceive YPD at 37°C as an environment whose inductive effect is already above the threshold required to switch to hyphal growth. Second, opaque phase *set3*Δ/Δ mutants grow predominantly as pseudohyphae on Lee's medium at 25°C as opposed to *set3*Δ/Δ white cells as well as wild type white or opaque cells (Figure 1). In this system, however, Lee's medium at 25°C already represents an environmental scenario close to the threshold point, which is supported by the fact that Lee's medium at 37°C drives hyphal growth [30], [37], [48]. Why do *set3*Δ/Δ cells filament only in the opaque but not in the white phase? Opaque cells express *EFG1* at lower levels than white cells [23], because in opaque cells *EFG1* is repressed directly by the master opaque regulator Wor1, and indirectly by the transcription factor Czf1 [22]. Therefore, filamentation of opaque *set3*Δ/Δ cells is most probably resulting from the increased cAMP/PKA pathway sensitivity due to the lack of Set3C, and the inhibition of the *EFG1* locus by Wor1 and Czf1 mimicking the effect of the *EFG1*-autoinhibitory loop (the latter being absent in the white phase). Notably, Lee's medium at 25°C is not a strong enough signal to induce true hyphal growth. This is in complete agreement with a recently proposed hypothesis, according to which weak filament-inducing signals turn on a set of genes required for pseudohyphal growth, while a stronger signal of the same nature turns on a set of additional genes triggering hyphal growth [49].

At which level of the pathway does the Set3C act on the molecular level? Recently, it was shown that *C. albicans* Efg1 binds to the promoters of filament-specific genes in both yeast and hyphal cells, and recruits the NuA4 histone acetyltransferase complex and the Swi/Snf chromatin remodeling complex upon hyphal induction [50]. Given that the *S. cerevisiae* Set3C possesses histone deacetylase activity [26], and that all subunits of the complex show strong evolutionary conservation (Table 1), it seems reasonable to propose that Set3C interferes with Efg1-dependent gene expression in *C. albicans* by effecting the chromatin status at Efg1 target loci. On the other hand, the PKA hyperactivity in *set3*Δ/Δ cells as judged from trehalose quantification and PKA activity assays (Figure 6D and 6E) apparently contradicts this notion, and implies that the Set3C acts either at the level or upstream of PKA (see Figure 6A). Nevertheless, a chromatin-based regulatory effect at Efg1 target loci would still be possible, if Efg1 coordinated a feed-back mechanism affecting the activity of PKA. We are developing molecular tools to address these possibilities directly. In this context, it is interesting to note that in a previous study, we found the histone methyltransferase *SET1* to be epistatic to *SET3* or *HOS2* to regulate white-opaque switching in *C. albicans* [25]. We interpreted these data in a way that the PHD domain of Set3 recognizes methylation marks generated by Set1 at its target loci. Indeed, such a recognition was subsequently demonstrated in *S. cerevisiae, in vivo* [51]. Consequently, since deletion of *SET1* and *SET3* showed a synergistic effect in repressing filamentation (Figure 3A), it is reasonable to hypothesize that at genetic loci where Set3C antagonizes filamentation, it does not depend on the methylation marks generated by Set1. Alternatively, it is also possible that the Set3C targets as yet unknown transcription factor(s), rather than histone proteins. This will have to be explored in more detail in future studies.

### Adenine as a morphogenetic signal

Along with serum and nitrogen-limitation, filamentation of *C. albicans* can be triggered by numerous specific stimuli, including human hormones, N-acetylglucosamine and bacterial peptidoglycans [4], [52]. We identify adenine as a potential signal that also has a modulatory effect on the yeast-filament conversion *in vitro*. Namely, exogenous adenine attenuated the hyperfilamentation phenotypes of *set3*Δ/Δ cells and suppressed the upregulation of filament-specific genes (Figure 5 and S4). Similar to the Set3C, adenine may also influence cAMP/PKA dependent signaling events in a negative way, since cAMP supplementation in the presence of adenine rescued the phenotype of *set3*Δ/Δ cells (Figure 6F). Since adenine in itself did not have an influence on the morphogenesis or marker gene expression in wild type cells (Figure 5), it appears likely that its importance as a metabolic factor is limited to specific conditions *in vivo*, which are partially mimicked by the disruption of the Set3C. In this context, it is interesting to note that limitation of nicotinic acid (like adenine, a NAD precursor) in a urinary tract infection model regulates cell adhesion through a chromatin-dependent mechanism in the fungal pathogen *Candida glabrata* [53].

In summary, although the specific nature of the adenine signal will have to be explored in further studies, it provides an additional argument that both white and opaque filamentation phenotypes of *set3*Δ/Δ mutants are caused by an interference with the same genetic mechanism. In addition, we have shown earlier that exogenous adenine also modulates opaque-to-white transition [25], which, together with the results provided here, indicate the need to study the role of purine-metabolism in the regulation of *C. albicans* morphogenesis.

### Morphogenesis as a virulence factor

Most human fungal pathogens including *Candida albicans, Cryptococcus neoformans, Blastomyces dermatitidis* or *Histoplasma capsulatum* are dimorphic. Therefore, morphogenesis has been extensively studied as a potential virulence trait [2]. For example, locking *Candida albicans* cells in the yeast form by the combined deletion of *CPH1* and *EFG1* abolishes virulence [9]. Likewise, locking cells in a filamentous form by deleting *TUP1* had a similar effect [11]. These data led to the general view that virulence is determined by the ability to change morphologies rather than the individual growth forms *per se*. Although this view is widely accepted, supporting evidence remains indirect, because the mutants tested so far are either locked in a specific growth form or display pleiotropic alterations of other cellular functions unrelated to morphogenesis. For instance, deletion of the transcriptional repressor *NRG1* locks cells in a filamentous state and *nrg1*Δ/Δ cells are avirulent [14]. Deletion of the hypha-specific cyclin *HGC1* locks cells in the yeast form and the *hgc1*Δ/Δ mutant is also avirulent [54]. Deletion of the transcriptional repressor *SSN6* permits yeast and pseudohyphal growth, but not true hyphal growth. Lack of *SSN6* affects generation time, and *ssn6*Δ/Δ cells are avirulent [33]. The most direct evidence addressing the contribution of a specific growth form to virulence came from two recent studies on the transcription factors *UME6* and *NRG1.* The ectopic overexpression of *UME6* converted cells to the hyphal growth mode *in vivo* rendering cells hypervirulent [49]. Conversely, ectopic overexpression of *NRG1* inhibited hyphal growth *in vivo* causing attenuated virulence [55]. These studies demonstrated the pivotal role of the hyphal morphology during infection.

In this study we describe a novel phenotype caused by the ablation of key components of the Set3C. The *C. albicans set3*Δ/Δ mutant is able to maintain both normal yeast and filamentous growth modes but is hyperfilamenting both *in vitro* and *in vivo*. Strikingly, the s*et3*Δ/Δ mutant shows attenuated virulence in a murine systemic infection model. However, we cannot rule out that loss of *SET3* alters expression of other yet unknown virulence genes whose functions are unrelated to morphogenesis. We are currently performing whole genome microarray analyses to address this possibility. Nevertheless, the virulence defect of s*et3*Δ/Δ cells *in vivo* as yet appears to highlight the importance of maintaining the yeast phase growth during certain stages of dissemination in the host. Since mice infected with s*et3*Δ/Δ cells recover from the infection more efficiently than mice infected with wild type cells, it appears that the adequate timing for filamentation in a given niche is crucial for full virulence. The ability to maintain the unicellular yeast morphology in host environments being important for virulence is not surprising, since several pleiomorphic pathogens are virulent mainly in the yeast form. For instance, both in the case of *Histoplasma capsulatum* and *Cryptococcus neoformans*, yeast phase cells are required for human infections [2], [56].

Regardless of other possible defects caused by impaired Set3C function, targeting the complex with specific deacetylase inhibitors can have a unique therapeutic potential. In fact, impairing the morphogenetic ability has been postulated as a promising area for antifungal drug discovery [55]. Trichostatin A (TSA), a known histone deacetylase inhibitor was recently shown to alter drug sensitivity and to inhibit serum-induced morphogenesis of some *C. albicans* strains [57], [58]. In addition, a novel fungal Hos2-inhibitor has entered clinical trials to prove therapeutic potential used in combination with the antifungal fluconazole [59]. Here, we show that TSA is a potent trigger of the yeast-to-hyphae conversion of *C. albicans*. Most importantly, out of all putative histone deacetylase genes, only the deletion of Hos2, the second catalytic subunit of the Set3C can phenocopy TSA treatment, providing compelling evidence that inhibition of the Set3C by TSA is causing the morphogenetic defects. Currently, we are investigating a possible therapeutic potential of TSA and related compounds to modulate morphogenesis and virulence *in vivo*.

In conclusion, we establish the Set3C as a novel key regulator of morphogenesis in *C. albicans*. We propose that compromising the complex activity leads to a “threshold shift” in the susceptibility to morphogenetic signals, and a consequent virulence defect. Since the complex appears well conserved from yeast to mammals [60], it will be fascinating to investigate the roles of the Set3C in the morphogenesis and virulence of other fungal pathogens. Indeed, the homologues of *SET3* and *HOS2* have recently been indentified as factors enhancing virulence of the human fungal pathogen *Cryptococcus neoformans* [61]. Taken together, our results emphasize the role of morphogenesis as a virulence factor and highlight the role of chromatin in regulating the activity of signaling pathways to orchestrate developmental changes in a simple eukaryotic model system.

## Materials and Methods

### Media and growth conditions

Rich medium (YPD) and complete synthetic medium (SD) was prepared as previously described [41]. Modified Lee's medium was prepared as described [48]. Unless indicated otherwise, *MTL* heterozygous and homozygous strains were routinely grown at 30°C and 25°C, respectively. GlcNAc, adenine and cAMP were purchased from Sigma.

### Strain construction

The complete list of *C. albicans* strains, primers and plasmids used in this study are listed in Supplementary Tables S1, S2 and S3, respectively. All strains were derived from SN152 [62], a leucine, histidine, arginine auxotrophic derivative of the clinical isolate SC5314 [63]. *SET3, HOS2, MKC1, CDC35, TPK1, TPK2, SNT1* and *SIF2* were deleted in SN152 using the fusion PCR strategy with the *C.m.LEU2* and *C.d.HIS1* markers [62]. The *cph1*Δ/Δ strain (JKC19) has been previously described [8]. All *MTL* **a**/**a** strains were constructed in the DHCA202 background, which is a *MTL* **a**/**a** derivative of SN152 [25]. The deletion mutants *set3*Δ/Δ, *hos2*Δ/Δ, *set1*Δ/Δ, *hst1*Δ/Δ, *efg1*Δ/Δ *set3*Δ/Δ, *efg1*Δ/Δ *hos2*Δ/Δ, *set1*Δ/Δ *set3*Δ/Δ and *set1*Δ/Δ *hos2*Δ/Δ in the DHCA202 background were described earlier [25]. *SET3* was deleted in the JKC19 and the *MTL* **a**/**a** *hst1*Δ/Δ strains to create the *cph1*Δ/Δ *set3*Δ/Δ and *hst1*Δ/Δ *set3*Δ/Δ mutants, respectively, using the pDH104 plasmid [25] linearized by PvuI restriction. *HOS2* was deleted in the JKC19 background to create the *cph1*Δ/Δ *hos2*Δ/Δ mutant using the pDH102 plasmid [25] linearized by digestion with PvuI. *SET3* was deleted in the *mkc1*Δ/Δ background using the fusion PCR strategy with the *C.d.ARG4* marker and the linearized pDH104 plasmid to create the *mkc1*Δ/Δ *set3*Δ/Δ strain. *CDC35, TPK1* and *TPK2* were deleted in the *set3*Δ/Δ background using the fusion PCR strategy with the *C.d.ARG4* and *SAT1* markers to create the *cdc35*Δ/Δ *set3*Δ/Δ, *tpk1*Δ/Δ *set3*Δ/Δ and *tpk2*Δ/Δ *set3*Δ/Δ strains. Except for the *cph1*Δ/Δ *set3*Δ/Δ, *cph1*Δ/Δ *hos2*Δ/Δ and *hst1*Δ/Δ *set3*Δ/Δ, *mkc1*Δ/Δ *set3*Δ/Δ *cdc35*Δ/Δ *set3*Δ/Δ and *tpk1*Δ/Δ *set3*Δ/Δ double mutants, at least two independent homozygous deletion strains were constructed from independent heterozygote isolates. Transformation was performed via electroporation as described [64]. Genomic integration events were verified with PCR and Southern blot analyses.

Gene complementation mutants were constructed using three approaches. In the *MTL* **a**/α *set3*Δ/Δ strains and the *MTL* **a**/**a** *hst1*Δ/Δ mutant, the *SET3* ORF was reintegrated into its endogenous locus using the pDH112 complementation plasmid [25] linearized by PvuI digestion. In the *MTL* **a**/**a** *set3*Δ/Δ strains, the *SET3* ORF was integrated into one allele of the *RP10* locus. To target the *RP10* locus, 5′ and 3′ homology regions corresponding to about +/−1kb up- and downstream of the start and stop codon of the *RP10* ORF, respectively, were amplified from SC5314 genomic DNA. The upstream fragment was cloned using the HindIII and BamHI restriction sites; the downstream fragment was cloned using the SacI and SacII sites into the pAG36 [65] plasmid harboring a nourseothricin acetyltransferase (*NAT1*) resistance marker, to yield the plasmid pRP53. The *SET3* ORF with its endogenous promoter was cloned into pRP53 using ApaI and NheI restriction sites introduced into pRP53 on the 3′ primer of the upstream RP10 cloning fragment, to create the complementation vector p7221. The pRP53 empty vector and the p7221 integration constructs were linearized by AgeI-digestion prior to transformation. Gene complementation construct for the *HOS2* ORF was created using the *SAT1* marker cassette of the plasmid pSFS2A and the fusion PCR strategy exactly as described [25]. Transformation was performed via electroporation as described [64]. Correct genomic integration of the complementation plasmids was verified by PCR analysis.

### Colony morphology analysis and microscopy

Colony morphology was analyzed using a Discovery V12 Stereoscope equipped with an Axiocam MR5 camera (Zeiss) Microscopic analysis was performed using an Olympus IX81 microscope equipped with a Hamamatsu Orca ER camera (Olympus). For fluorescence microscopy, cells were fixed in 70% ethanol for five minutes, washed three times with distilled water, stained with 10 µM Calcofluor White and 1 µg/ml DAPI (4′,6-Diamidino-2-phenylindole dihydrochloride) for five minutes, washed three times with distilled water and deposited onto glass slides.

### Generation time analysis

Strains were streaked from −80°C frozen stocks on YPD agar plates and incubated three days at 30°C. Single colonies were inoculated in liquid YPD and grown overnight, diluted to an OD_600_ of 1–2 and incubated three hours at 30°C. Cultures were diluted into fresh YPD to an OD_600_ of 0.1–0.3; subsequently, OD_600_ values were measured every hour. The generation times were calculated by fitting an exponential function on the exponential parts of the growth curves using the Origin 6.1 software (MicroCal).

### RNA isolation and quantitative qRT-PCR

Strains were streaked from −80°C frozen stocks onto YPD agar plates and incubated three days at 30°C. Opaque phase cultures, as well as the white isolates of the corresponding genotypes were streaked from −80°C frozen stocks on YPD agar plates containing 5 µg/ml Phloxin B and incubated three days at 25°C. Single colonies were suspended in distilled water and spread at low densities onto media indicated at each experiment, and incubated using conditions described for each experiment. Colonies (1–3) were scraped off plates and suspended in 500 µl TRI Reagent (Molecular Research Center). After addition of around 200 µl glass beads (425–600 µm, Sigma), cells were broken at 5 m/s for 45 seconds on a FastPrep instrument (MP Biomedicals). Tubes were centrifuged and the supernatant (around 300 µl) was transferred to a fresh tube, 500 µl TRI Reagent and 160 µl chloroform was added. After centrifugation at 14000 g for 15 min at 4°C, the aqueous phase was extracted once with phenol:chloroform:isoamylalcohol. RNA was precipitated in 70% ethanol at −20°C overnight, washed once with 70% ethanol and dissolved in distilled water. About 5–10 µg total RNA was treated with DNaseI (Fermentas). Subsequently, about 1–5 µg of total RNA was reverse-transcribed with the First Strand cDNA synthesis kit (Fermentas). cDNA amplification was monitored quantitatively by SYBR Green incorporation in a Realplex Mastercycler (Eppendorf) using the MesaGreen Master mix (Eurogentec). Amplification curves were analyzed using the Realplex Software (Eppendorf). Statistics analysis (Student's t-test) was performed in Excel (Microsoft).

### Western blot analysis

Colonies were scraped off plates and cells were washed three times with ice-cold distilled water. About 20 mg (±0.5 mg) wet weight of cells of each culture were measured and total cell extracts were prepared exactly as previously described [25]. For Western blot analysis, extracts from 0.5 mg wet cells were separated by SDS-PAGE. The phosphorylated forms of the Mkc1 and Cek1 MAP Kinases were detected with a phospho-p44/42 antibody (#9101, Cell Signaling). Loading controls were visualized using a monoclonal anti-tubulin antibody (DM1A, Sigma). Western blot experiments were repeated three times.

### Trehalose determination

Colonies were scraped off plates and cells were washed three times with ice-cold distilled water. About 20 mg (±0.5 mg) wet weight of cells of each culture were frozen in liquid nitrogen. For the separation of yeast and hyphal phases, the colonies were filtered through a 70 µm cell strainer (BD Falcon) prior to the washing steps. The pellets were resuspended in 0.5 ml of 0.25 M Na_2_CO_3_ per 20 mg of cells, boiled at 95°C for 20 min, and centrifuged at 14.000 g for 5 min. A 10 µl aliquot of the supernatant was neutralized by the addition of 6.5 µl 1M acetic acid. For each reaction, 5 µl of buffer T (300 mM NaAc, 30 mM CaCl_2_, pH 5.5) and 3 µl of porcine kidney trehalase was added (3.7 U/ml, Sigma), and the volume was adjusted to 43 µl with distilled water. Reactions were incubated at 37°C for 45 min. The glucose liberated was measured in 25 µl of each reaction using the glucose assay kit from Sigma according to the manufacturer's instructions.

### Protein Kinase A activity assay

Colonies were scraped off agar plates and about 100 mg (±50 mg) wet weight of cells of each culture was washed twice with ice-cold distilled water. For the separation of yeast and hyphal phases, the colonies were filtered through a 70 µm cell strainer (BD Falcon) prior to the washing steps. Crude cell extracts were prepared as previously described [66]. Briefly, cell pellets were resuspended in 250 µl of 10 mM sodium phosphate buffer (pH 6.8) containing 1 mM EGTA, 1 mM EDTA, 10 mM β-mercaptoethanol. Protease inhibitor tablets (Roche) were added prior to use. After resuspension, glass beads were added (425–600 µm, Sigma), and cells were broken at 4 m/s for 30 seconds on a FastPrep instrument (MP Biomedicals). Tubes were centrifuged twice at 14000 rpm at 4°C for 15 minutes and the supernatant was transferred to a fresh tube. Total protein concentrations were adjusted with the Bradford method, and extracts containing 10 µg of total protein were immediately used for the enzymatic assay. Total PKA activity was measured following the guidelines of the PepTag cAMP-dependent protein kinase assay kit (Promega) in a total volume of 50 µl, containing 20 mM Tris-HCl (pH 7.4), 10 mM MgCl_2_, 1 mM ATP and 2 µg of PepTag A1 Peptide. 10 µM cAMP and 250 µM H-89 (LC Laboratories) were added when indicated. Reactions were incubated at 30°C for 30 minutes, heat-inactivated at 95°C for 10 min and separated on a 0.8% agarose gel in 50 mM Tris (pH 8.0) buffer (Figure S5B). Quantification of the phosphorylated.

PepTag peptide fractions excised from the gels was performed by spectrophotometry according to the manufacturer's instructions.

### Virulence assays

Strains were streaked from −80°C frozen stocks on YPD agar plates and incubated two-three days at 30°C. Single colonies were inoculated in liquid YPD and grown until the mid-exponential growth phase (around OD_600_ 1). Cells were washed twice with PBS and the concentrations were adjusted with a hemocytometer. About 5*10^5^ cells were injected in 100–110 µl suspensions in PBS through the lateral tail vein into 6–8 week old male BALB/c mice. The mice all weighed between 17 and 20 grams and 10 mice per *C. albicans* genotype were used, except for the PBS control for which only three mice were injected. Survival was monitored over a three week period. Curves were plotted and statistical analysis (Log-rank test) was carried out using the Prism software (GraphPad). The virulence assay was repeated twice.

For histopathology, *C. albicans* cells were prepared and injected exactly as described for the survival experiments. Three 6–8 week old male BALB/c mice were injected per each *C. albicans* genotype, plus two mice were injected with PBS. Animals were sacrificed on Day 1 after the injection. Kidneys were fixed in 4% paraformaldehyde and embedded in paraffin. Serial sections (2 µm) were stained with Grocott staining using the Bio-Optica Kit according to the manufacturer's instructions. All animal experiments were performed according to the guidelines of the Austrian Ministry of Science and Research and were approved by the animal ethics committee of the Medical University Vienna under the protocol number BMWF-68.205/0233-II/10b/2009.

## Supporting Information

Figure S1 Additional phenotypes of the *set3*Δ/Δ mutant. (A) Colony morphology of wild type and *set3*Δ/Δ mutant strains on the indicated solid media. Both strains are *MTL* **a**/α strains. Scale bar corresponds to 2 mm. (B) Morphology of wild and type and *set3*Δ/Δ mutant strains in the indicated liquid media. Both strains are *MTL* **a**/α strains. Saturated overnight cultures (first line) were diluted 1:50 in the media indicated. Scale bar corresponds to 5μm.
(1.94 MB TIF)

Figure S2 Additional phenotypes of the *set3*Δ/Δ mutant (2). (A) Microscopic analysis of the colonies shown on Figure 2A. The wrinkled colony of *set3*Δ/Δ cells consists of a mixture of yeast cells and filaments. Scale bar corresponds to 5μm. (B) Microscopic analysis of the colonies shown on Figure 1A. The wrinkled colony of the opaque *MTL* **a**/**a** *set3*Δ/Δ cells consists of a mixture of yeast cells and filaments. Scale bar corresponds to 5μm. (C) The hyperfilamentation phenotype of white phase cells observed on YPD at 37°C is independent of *MTL*-zygosity. Colony images were taken after three days of incubation. All strains are *MTL* **a**/**a** strains. Scale bar corresponds to 2mm.
(1.14 MB TIF)

Figure S3 *SET3* and *HOS2* mRNA levels are unaltered upon serum induction. Quantitative Realtime PCR analysis was performed with cDNA samples derived from the colonies shown on Figure 2A. Transcript levels were normalized against the expression level of *RIP1*. qRT-PCR reactions were performed in triplicates and RNA isolated from two independent cultures were analyzed. Data are shown as mean + SD.
(0.09 MB TIF)

Figure S4 Additional expression profiles of *set3*- and *hos2*-mutants. For the experimental logic see Figure 4A. Quantitative Realtime PCR analysis was performed with cDNA samples derived from the colonies shown on Figures 1A and 3A. Transcript levels were normalized against the expression level of *RIP1*. qRT-PCR reactions were performed in triplicates and RNA isolated from two independent cultures were analyzed. Data are shown as mean + SD. FCS denotes YPD supplemented with 10% FCS. (A) The expression of *ECE1* is strongly induced in *set3*Δ/Δ and *hos2*Δ/Δ cells even on YPD at 37°C, which is a mild inducing stimulus for wild type cells. However, *ECE1* expression is abolished once *EFG1* is deleted both in wild type and *set3*Δ/Δ or *hos2*Δ/Δ cells. Double asterisk indicates statistical significance of P<0.01 between *set3*Δ/Δ and wild type or *hos2*Δ/Δ and wild type cells cultured under the same conditions (Student's t-test). (B) The expression profile of *FRE2* reveals at least three regulatory inputs. First, *FRE2* repressed by Tup1, because *tup1*Δ/Δ cells express *FRE2* in higher levels than wild type cells under all conditions tested. Second, *FRE2* is serum-induced in an *EFG1*-dependent manner. Third, *FRE2* expression is induced by serum by a mechanism other than *EFG1*-signalling. Most notably, the effect of *EFG1*-deletion appears epistatic to the *SET3* and *HOS*-deletions. (C) *DDR48* is repressed by Tup1, but not by Set3 or Hos2 under all conditions tested. (D) *ECE1* expression is strongly induced in opaque phase *set3*Δ/Δ cells, but the induction is suppressed by deletion of *EFG1* or by supplementing the medium with 100μg/ml adenine. Double asterisk indicates statistical significance of P<0.01 between *set3*Δ/Δ and wild cells of the same phase cultured under the same conditions (Student's t-test).
(0.57 MB TIF)

Figure S5 Additional trehalose and PKA activity measurements to link hyperreactivity of cAMP/PKA signalling to deletion of *SET3*. (A) Trehalose content of colonies grown on the indicated media. *set3*Δ/Δ and *hos2*Δ/Δ cells apparently contain less trehalose on FCS plates after three days, indicating high PKA activity. Asterisk indicates statistical significance of P<0.05 relative to wild type cells of the same phase cultured under identical conditions (Student's t-test). (B) Protein kinase activities of cell extracts derived from the indicated colonies. “T”: total, “Y”: yeast, “H”: hyphal fraction. The phosphorylated PepTag PKA substrate migrates towards the kathode, whereas the unphosphorylated form migrates towards the anode. The measured activity appears specific for PKA, since it is further inducible by addition of cAMP, and is inhibited by the H-89 inhibitor. 2ng of purified PKA of the cAMP-dependent protein kinase assay kit (Promega) was used as a control to titrate the inhibitor. The spectrophotometric quantification of the upper panel is found on Figure 6E.
(0.82 MB TIF)

Table S1 *C. albicans* strains used in this study.
(0.11 MB DOC)

Table S2 Oligonucleotide primers used in this study.
(0.09 MB DOC)

Table S3 Plasmids used in this study.
(0.04 MB DOC)
