# Supplementary figures and images for: The Set3/Hos2 Histone Deacetylase Complex Attenuates cAMP/PKA Signaling to Regulate Morphogenesis and Virulence of Candida albicans
Source: PLoS Pathog. 2010 May 13;6(5):e1000889. doi: 10.1371/journal.ppat.1000889 (PMC2869326; doi:10.1371/journal.ppat.1000889)

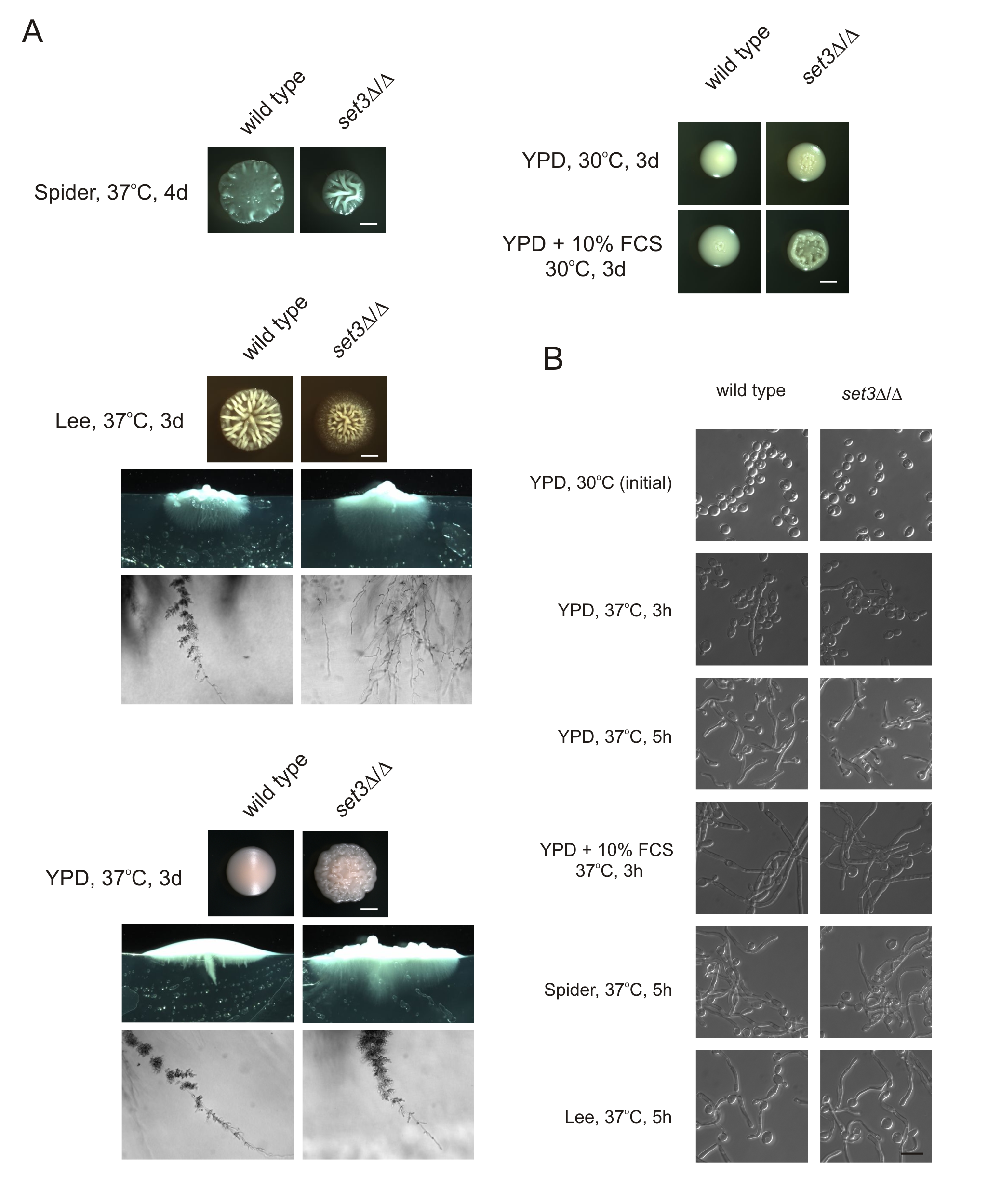

Supplement: Figure S1 — Additional phenotypes of the set3Δ/Δ mutant. (A) Colony morphology of wild type and set3Δ/Δ mutant strains on the indicated solid media. Both strains are MTL a/α strains. Scale bar corresponds to 2 mm. (B) Morphology of wild and type and set3Δ/Δ mutant strains in the indicated liquid media. Both strains are MTL a/α strains. Saturated overnight cultures (first line) were diluted 1:50 in the media indicated. Scale bar corresponds to 5μm. (1.94 MB TIF) [file ppat.1000889.s001.tif]

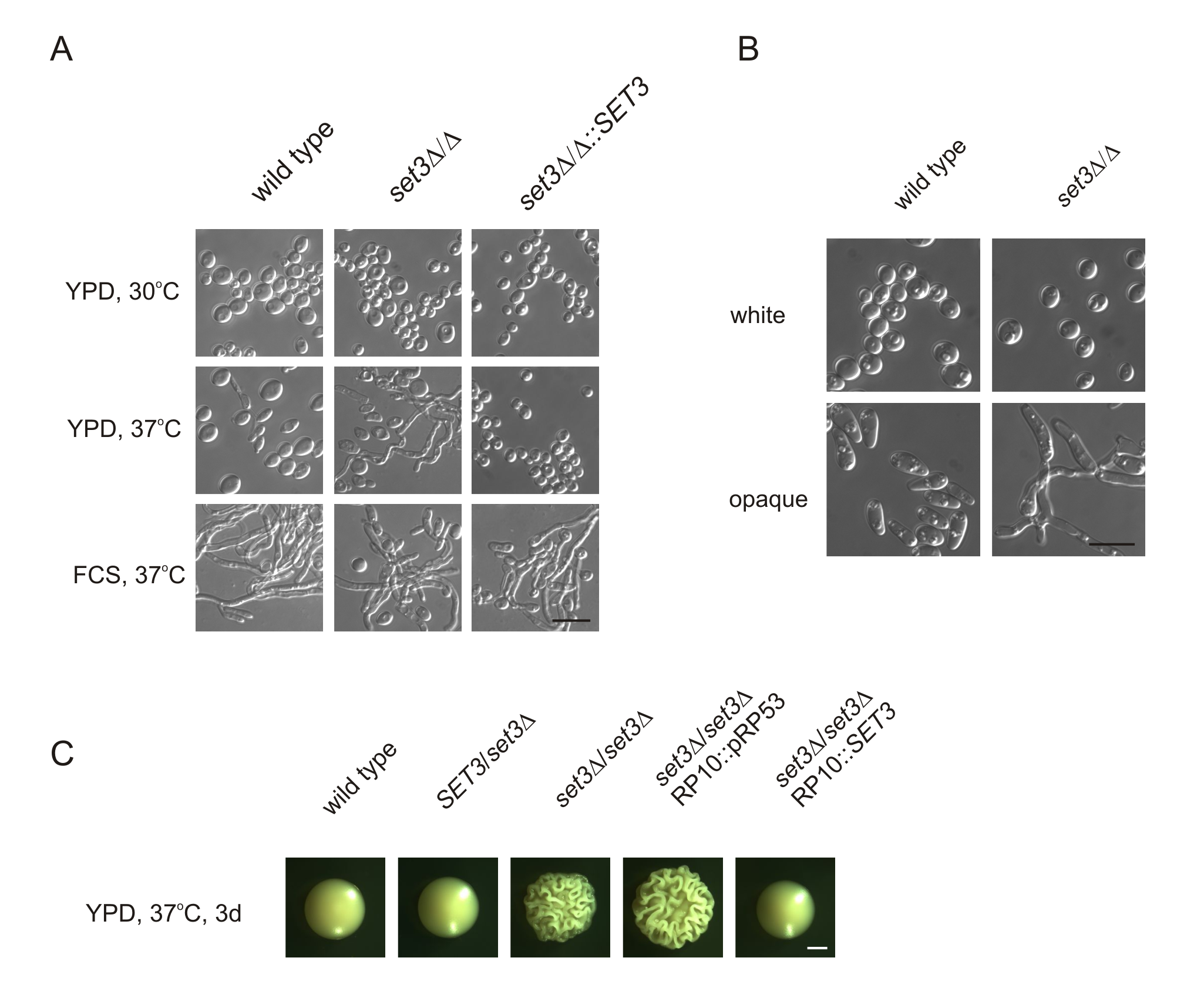

Supplement: Figure S2 — Additional phenotypes of the set3Δ/Δ mutant (2). (A) Microscopic analysis of the colonies shown on Figure 2A. The wrinkled colony of set3Δ/Δ cells consists of a mixture of yeast cells and filaments. Scale bar corresponds to 5μm. (B) Microscopic analysis of the colonies shown on Figure 1A. The wrinkled colony of the opaque MTL a/a set3Δ/Δ cells consists of a mixture of yeast cells and filaments. Scale bar corresponds to 5μm. (C) The hyperfilamentation phenotype of white phase cells observed on YPD at 37°C is independent of MTL-zygosity. Colony images were taken after three days of incubation. All strains are MTL a/a strains. Scale bar corresponds to 2mm. (1.14 MB TIF) [file ppat.1000889.s002.tif]

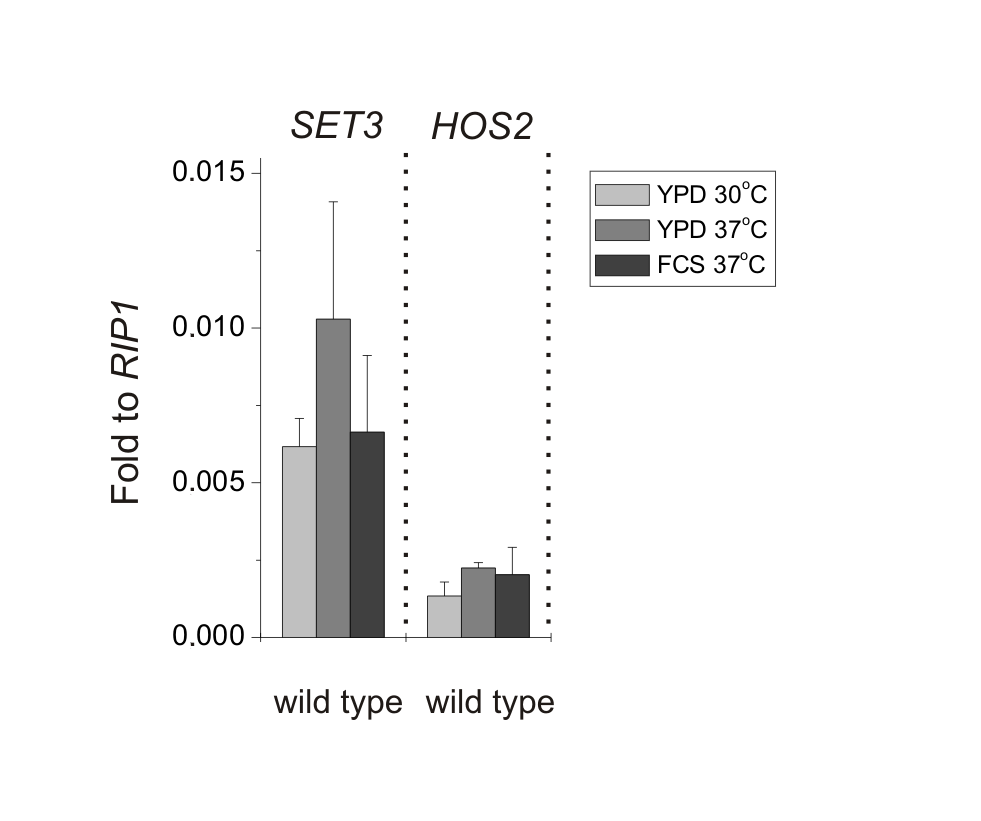

Supplement: Figure S3 — SET3 and HOS2 mRNA levels are unaltered upon serum induction. Quantitative Realtime PCR analysis was performed with cDNA samples derived from the colonies shown on Figure 2A. Transcript levels were normalized against the expression level of RIP1. qRT-PCR reactions were performed in triplicates and RNA isolated from two independent cultures were analyzed. Data are shown as mean + SD. (0.09 MB TIF) [file ppat.1000889.s003.tif]

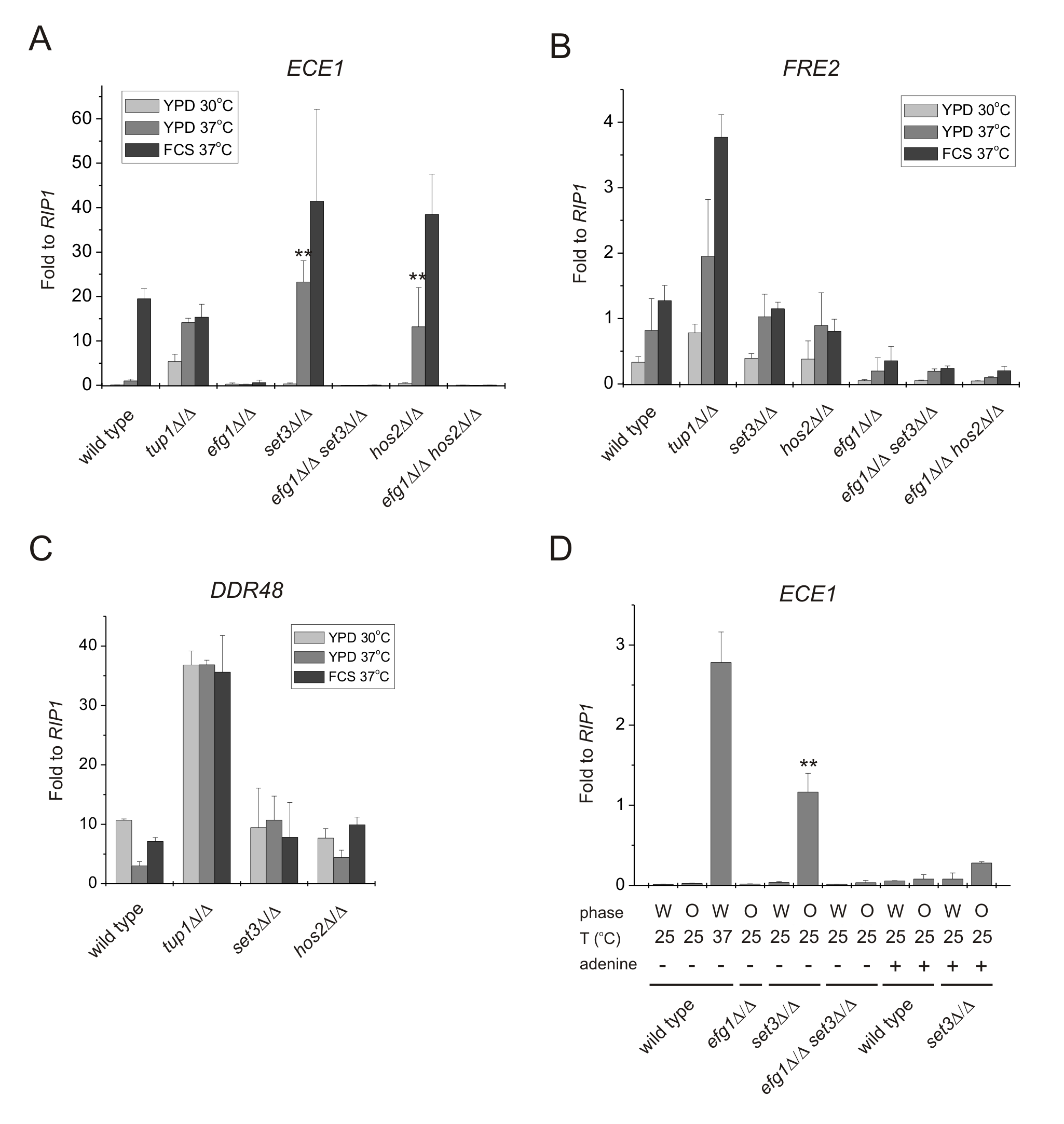

Supplement: Figure S4 — Additional expression profiles of set3- and hos2-mutants. For the experimental logic see Figure 4A. Quantitative Realtime PCR analysis was performed with cDNA samples derived from the colonies shown on Figures 1A and 3A. Transcript levels were normalized against the expression level of RIP1. qRT-PCR reactions were performed in triplicates and RNA isolated from two independent cultures were analyzed. Data are shown as mean + SD. FCS denotes YPD supplemented with 10% FCS. (A) The expression of ECE1 is strongly induced in set3Δ/Δ and hos2Δ/Δ cells even on YPD at 37°C, which is a mild inducing stimulus for wild type cells. However, ECE1 expression is abolished once EFG1 is deleted both in wild type and set3Δ/Δ or hos2Δ/Δ cells. Double asterisk indicates statistical significance of P<0.01 between set3Δ/Δ and wild type or hos2Δ/Δ and wild type cells cultured under the same conditions (Student's t-test). (B) The expression profile of FRE2 reveals at least three regulatory inputs. First, FRE2 repressed by Tup1, because tup1Δ/Δ cells express FRE2 in higher levels than wild type cells under all conditions tested. Second, FRE2 is serum-induced in an EFG1-dependent manner. Third, FRE2 expression is induced by serum by a mechanism other than EFG1-signalling. Most notably, the effect of EFG1-deletion appears epistatic to the SET3 and HOS-deletions. (C) DDR48 is repressed by Tup1, but not by Set3 or Hos2 under all conditions tested. (D) ECE1 expression is strongly induced in opaque phase set3Δ/Δ cells, but the induction is suppressed by deletion of EFG1 or by supplementing the medium with 100μg/ml adenine. Double asterisk indicates statistical significance of P<0.01 between set3Δ/Δ and wild cells of the same phase cultured under the same conditions (Student's t-test). (0.57 MB TIF) [file ppat.1000889.s004.tif]

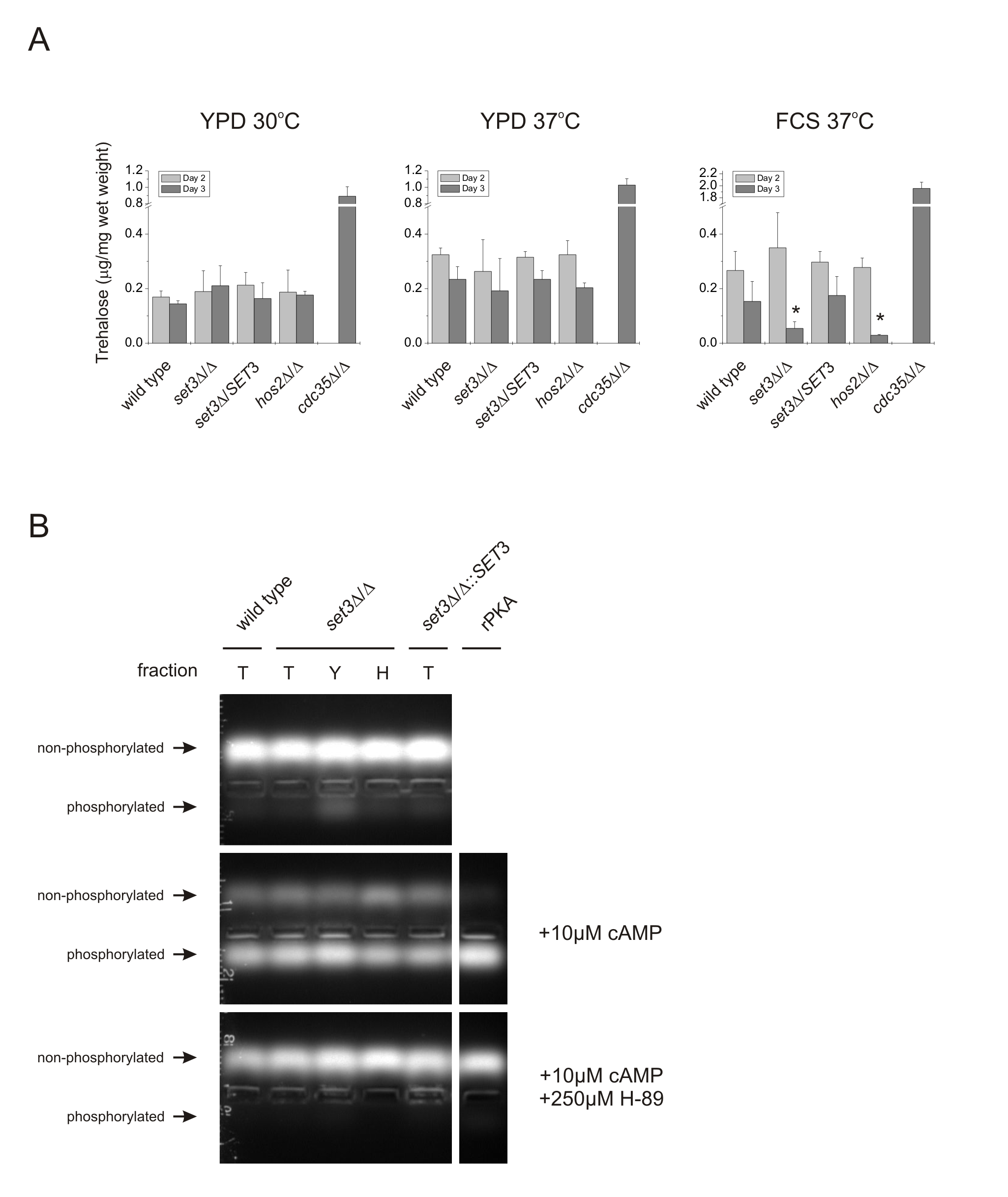

Supplement: Figure S5 — Additional trehalose and PKA activity measurements to link hyperreactivity of cAMP/PKA signalling to deletion of SET3. (A) Trehalose content of colonies grown on the indicated media. set3Δ/Δ and hos2Δ/Δ cells apparently contain less trehalose on FCS plates after three days, indicating high PKA activity. Asterisk indicates statistical significance of P<0.05 relative to wild type cells of the same phase cultured under identical conditions (Student's t-test). (B) Protein kinase activities of cell extracts derived from the indicated colonies. “T”: total, “Y”: yeast, “H”: hyphal fraction. The phosphorylated PepTag PKA substrate migrates towards the kathode, whereas the unphosphorylated form migrates towards the anode. The measured activity appears specific for PKA, since it is further inducible by addition of cAMP, and is inhibited by the H-89 inhibitor. 2ng of purified PKA of the cAMP-dependent protein kinase assay kit (Promega) was used as a control to titrate the inhibitor. The spectrophotometric quantification of the upper panel is found on Figure 6E. (0.82 MB TIF) [file ppat.1000889.s005.tif]
