# Supplementary material for: The Set3/Hos2 Histone Deacetylase Complex Attenuates cAMP/PKA Signaling to Regulate Morphogenesis and Virulence of Candida albicans
Source: PLoS Pathog. 2010 May 13;6(5):e1000889. doi: 10.1371/journal.ppat.1000889 (PMC2869326; doi:10.1371/journal.ppat.1000889)
Supplement: Table S1 — C. albicans strains used in this study. (0.11 MB DOC) [file ppat.1000889.s006.doc]

**Table S1.** ***C. albicans*** **strains used in this study**

| **Description** | **Name** | ***MTL*** | **Parent** | **Genotype** | **Reference** | **Figure/Table** |
| --- | --- | --- | --- | --- | --- | --- |
| Wild type | SC5314 | **a**/α | Clinical isolate |  | [1] |  |
| Wild type | SN152 | **a**/α | SC5314 | *arg4*Δ/*arg4*Δ *his1*Δ/*his1*Δ *leu2*Δ/*leu2*Δ *URA3*/*ura3*Δ::*λimm^434^* *IRO1*/*iro1*Δ::*λimm^434^* | [2] |  |
| Wild type | CAIF100 | **a**/α | SN152 | *arg4*Δ/*arg4*Δ *his1*Δ/*his1*Δ::*C.d.HIS1 leu2*Δ/*leu2*Δ::*C.m.LEU2 URA3*/*ura3*Δ::*λimm^434^* *IRO1*/*iro1*Δ::*λimm^434^* | [3] | Figure 2, 3, 4, 5, 6, 7, S1, S2, S3, S4, S5 |
| Wild type | DHCA202 | **a**/**a** | SN152 | *arg4*Δ/*arg4*Δ *his1*Δ/*his1*Δ *leu2*Δ/*leu2*Δ *URA3*/*ura3*Δ::*λimm^434^* *IRO1*/*iro1*Δ::*λimm^434^* | [4] | Figure 1, 2, 3, 5, S2, S4 |
| *SET3/set3*Δ | DHCA401  DHCA403 | **a**/α | SN152 | *SET3*/*set3*Δ::*C.d.HIS1* | This study | Figure 2, 6, 7, S5 |
| *set3*Δ/Δ (#1)  (#2) | DHCA402  DHCA404 | **a**/α | SN152 | *set3*Δ::*C.d.HIS1*/*set3*Δ::*C.m.LEU2* | This study | Figure 2, 3, 4, 5, 6, 7, S1, S2, S4, S5 |
| *set3*Δ/Δ::*SET3* | DHCA405  DHCA406 | **a**/α | SN152 | *set3*Δ::*C.m.LEU2*/*set3*Δ::*SET3::*FRT | This study | Figure 2, 6, 7, S2, S5 |
| *SET3/set3*Δ | DHCA241  DHCA243 | **a**/**a** | DHCA202 | *SET3*/*set3*Δ::*C.d.HIS1* | [4] | Figure 1 |
| *set3*Δ/Δ | DHCA242  DHCA244 | **a**/**a** | DHCA202 | *set3*Δ::*C.d.HIS1*/*set3*Δ::*C.m.LEU2* | [4] | Figure 1, 2, 3, 5, S2, S4 |
| *set3*Δ/Δ  *RP10::*pRP53 | DHCA417 | **a**/**a** | DHCA202 | *set3*Δ::*C.d.HIS1*/*set3*Δ::*C.m.LEU2*  *RP10::pRP53* | This study | Figure 1 |
| *set3*Δ/Δ  *RP10::SET3* | DHCA418  DHCA419 | **a**/**a** | DHCA202 | *set3*Δ::*C.d.HIS1*/*set3*Δ::*C.m.LEU2*  *RP10::p7221* | This study | Figure 1, 5 |
| *hst1*Δ/Δ | DHCA250  DHCA252 | **a**/**a** | DHCA202 | *hst1*Δ::*C.d.HIS1*/*hst1*Δ::*C.m.LEU2* | [4] | Figure 1, 2 |
| *hos2*Δ/Δ | DHCA406 | **a**/α | SN152 | *hos2*Δ::*C.d.HIS1*/*hos2*Δ::*C.m.LEU2* | This study | Figure 2, 3, 4, 6, 7, S4, S5 |
| *hos2*Δ/Δ | DHCA246  DHCA248 | **a**/**a** | DHCA202 | *hos2*Δ::*C.d.HIS1*/*hos2*Δ::*C.m.LEU2* | [4] | Figure 1, 1 |
| *hos2*Δ/Δ::*HOS2* | DHCA420 | **a**/α | SN152 | *hos2*Δ::*C.m.LEU2*/*hos2*Δ::*HOS2-SAT1* | This study | Figure 2 |
| *snt1*Δ/Δ | DHCA452 | **a**/α | SN152 | *snt1*Δ::*C.d.HIS1*/*snt1*Δ::*C.m.LEU2* | This study | Figure 2 |
| *sif2*Δ/Δ | DHCA454 | **a**/α | SN152 | *sif2*Δ::*C.d.HIS1*/*sif2*Δ::*C.m.LEU2* | This study | Figure 2 |
| *set3*Δ/Δ *hst1*Δ/Δ | DHCA410 | **a**/**a** | DHCA202 | *hst1*Δ::*C.d.HIS1*/*hst1*Δ::*C.m.LEU2*  *set3*Δ::FRT/*set3*Δ::FRT | This study | Figure 1, 2 |
| *hda1*Δ/Δ | DHCA232 | **a**/**a** | DHCA202 | *hda1*Δ::*C.d.HIS1*/*hda1*Δ::*C.m.LEU2* | [4] | Figure 2 |
| *rpd31*Δ/Δ | DHCA238 | **a**/**a** | DHCA202 | *rpd31*Δ::*C.d.HIS1*/*rpd31*Δ::*C.m.LEU2* | [4] | Figure 2 |
| *rpd3*Δ/Δ | DHCA360 | **a**/**a** | DHCA202 | *rpd3*Δ::*C.d.HIS1*/*rpd3*Δ::*C.m.LEU2* | This study | Figure 2 |
| *hst2*Δ/Δ | DHCA256 | **a**/**a** | DHCA202 | *hst2*Δ::*C.d.HIS1*/*hst2*Δ::*C.m.LEU2* | [4] | Figure 2 |
| *sir2*Δ/Δ | DHCA254 | **a**/**a** | DHCA202 | *sir2*Δ::*C.d.HIS1*/*sir2*Δ::*C.m.LEU2* | [4] | Figure 2 |
| *hos1*Δ/Δ | DHCA274 | **a**/**a** | DHCA202 | *hos1*Δ::*C.d.HIS1*/*hos1*Δ::*C.m.LEU2* | [4] | Figure 2 |
| *hos3*Δ/Δ | DHCA280 | **a**/**a** | DHCA202 | *hos3*Δ::*C.d.HIS1*/*hos3*Δ::*C.m.LEU2* | [4] | Figure 2 |
| *cph1*Δ/Δ | JKC19 | **a**/α | SC5314 | *ura3*Δ*::λimm^434^*/*ura3*Δ*::λimm^434^* *cph1*Δ::*hisG*/*cph1*Δ::*hisGURA3hisG* | [5] | Figure 3 |
| *cph1*Δ/Δ *set3*Δ/Δ | DHCA414 | **a**/α | JKC19 | *ura3*Δ*::λimm^434^*/*ura3*Δ*::λimm^434^* *cph1*Δ::*hisG*/*cph1*Δ::*hisGURA3hisG*  *set3*Δ::FRT/*set3*Δ::FRT | This study | Figure 3 |
| *cph1*Δ/Δ *hos2*Δ/Δ | DHCA416 | **a**/α | JKC19 | *ura3*Δ*::λimm^434^*/*ura3*Δ*::λimm^434^* *cph1*Δ::*hisG*/*cph1*Δ::*hisGURA3hisG*  *hos2*Δ::FRT/*hos2*Δ::FRT | This study | Figure 3 |
| *efg1*Δ/Δ | DHCA216 | **a**/α | SC5314 | *efg1*Δ::FRT/*efg1*Δ::FRT | [4] | Figures 3, 4, S4 |
| *efg1*Δ/Δ | DHCA212 | **a**/**a** | DHCA202 | *efg1*Δ::FRT/*efg1*Δ::FRT | [4] | Figures 3, 5, S4 |
| *efg1*Δ/Δ *hos2*Δ/Δ | DHCA302  DHCA304 | **a**/**a** | DHCA202 | *hos2*Δ::*C.d.HIS1*/*hos2*Δ::*C.m.LEU2 efg1*Δ::FRT/*efg1*Δ::FRT | [4] | Figures 3, 4, S4 |
| *efg1*Δ/Δ *set3*Δ/Δ | DHCA298  DHCA300 | **a**/**a** | DHCA202 | *set3*Δ::*C.d.HIS1*/*set3*Δ::*C.m.LEU2 efg1*Δ::FRT/*efg1*Δ::FRT | [4] | Figures 3, 4, 5, S4 |
| *set1*Δ/Δ | DHCA226  DHCA228 | **a**/**a** | DHCA202 | *set1*Δ::*C.d.HIS1*/*set1*Δ::*C.m.LEU2* | [4] | Figure 3 |
| *set1*Δ/Δ *hos2*Δ/Δ | DHCA326  DHCA328 | **a**/**a** | DHCA202 | *set1*Δ::*C.d.HIS1*/*set1*Δ::*C.m.LEU2 hos2*Δ::FRT/*hos2*Δ::FRT | [4] | Figure 3 |
| *set1*Δ/Δ *set3*Δ/Δ | DHCA330 | **a**/**a** | DHCA202 | *set1*Δ::*C.d.HIS1*/*set1*Δ::*C.m.LEU2 set3*Δ::FRT/*set3*Δ::FRT | [4] | Figure 3 |
| *cdc35*Δ/Δ | DHCA442 | **a**/α | SN152 | *cdc35*Δ::*C.d.HIS1*/*cdc35*Δ::*C.m.LEU2* | This study | Figure 6 |
| *cdc35*Δ/Δ *set3*Δ/Δ | DHCA444 | **a**/α | SN152 | *set3*Δ::*C.d.HIS1*/*set3*Δ::*C.m.LEU2*  *cdc35*Δ::*C.d.ARG4*/*cdc35*Δ::*SAT1* | This study | Figure 6 |
| *mkc1*Δ/Δ | CA_CG2 | **a**/α | SN152 | *mkc1*Δ::*C.d.HIS1*/*mkc1*Δ::*C.m.LEU2* | This study | Figure 6 |
| *mkc1*Δ/Δ *set3*Δ/Δ | DHCA448 | **a**/α | SN152 | *mkc1*Δ::*C.d.HIS1*/*mkc1*Δ::*C.m.LEU2*  *set3*Δ::*C.d.ARG4*/*set3*Δ::*pDH104* | This study | Figure 6 |
| *tpk1*Δ/Δ | DHCA456 | **a**/α | SN152 | *tpk1*Δ::*C.d.HIS1*/*tpk1*Δ::*C.m.LEU2* | This study | Figure 6 |
| *tpk1*Δ/Δ *set3*Δ/Δ | DHCA460 | **a**/α | SN152 | *set3*Δ::*C.d.HIS1*/*set3*Δ::*C.m.LEU2*  *tpk1*Δ::*C.d.ARG4*/*tpk1*Δ::*SAT1* | This study | Figure 6 |
| *tpk2*Δ/Δ | DHCA458 | **a**/α | SN152 | *tpk2*Δ::*C.d.HIS1*/*tpk2*Δ::*C.m.LEU2* | This study | Figure 6 |
| *tpk2*Δ/Δ *set3*Δ/Δ | DHCA462 | **a**/α | SN152 | *set3*Δ::*C.d.HIS1*/*set3*Δ::*C.m.LEU2*  *tpk2*Δ::*C.d.ARG4*/*tpk2*Δ::*SAT1* | This study | Figure 6 |

*In case of multiple deletion mutants per genotype, the data displayed in Figures 1-9 were derived from assays performed with the mutant of the lower index number (as described at the Materials and Methods).

**SUPPLEMENTARY REFERENCES**

1. Gillum AM, Tsay EY, Kirsch DR (1984) Isolation of the *Candida albicans* gene for orotidine-5'-phosphate decarboxylase by complementation of *S. cerevisiae* *ura3* and *E. coli* pyrF mutations. Mol Gen Genet 198: 179-182.

2. Noble SM, Johnson AD (2005) Strains and strategies for large-scale gene deletion studies of the diploid human fungal pathogen *Candida albicans*. Eukaryot Cell 4: 298-309.

3. Frohner IE, Bourgeois C, Yatsyk K, Majer O, Kuchler K (2008) *C. albicans* Cell Surface Superoxide Dismutases Degrade Host-Derived Reactive Oxygen Species to Escape Innate Immune Surveillance. Mol Microbiol.

4. Hnisz D, Schwarzmuller T, Kuchler K (2009) Transcriptional loops meet chromatin: a dual-layer network controls white-opaque switching in *Candida albicans*. Mol Microbiol 74: 1-15.

5. Liu H, Kohler J, Fink GR (1994) Suppression of hyphal formation in *Candida albicans* by mutation of a *STE12* homolog. Science 266: 1723-1726.
