# Supplementary material for: The Set3/Hos2 Histone Deacetylase Complex Attenuates cAMP/PKA Signaling to Regulate Morphogenesis and Virulence of Candida albicans
Source: PLoS Pathog. 2010 May 13;6(5):e1000889. doi: 10.1371/journal.ppat.1000889 (PMC2869326; doi:10.1371/journal.ppat.1000889)
Supplement: Table S2 — Oligonucleotide primers used in this study. (0.09 MB DOC) [file ppat.1000889.s007.doc]

**Supplementary Table 2.** **Oligonucleotide primers used in this study**

| **Name** | **Sequence (5´- 3´)*** | **Reference** |
| --- | --- | --- |
| **Gene deletion constructs based on fusion PCR strategy [2]** | |  |
| 55_CA7221 | ATTGTACGAAGAAGCGGAGC | [1] |
| 53_CA7221 | cacggcgcgcctagcagcggCATTAAAATAAACACTTATAAAGACTACTATC | [1] |
| 35_CA7221 | gtcagcggccgcatccctgcTAGTTTTTTGTTTAGAGTTTGTATATTG | [1] |
| 33_CA7221 | CAAAAGGACAATCAATTGGATG | [1] |
| 55_CA5377 | CAGAATCTTGACCTGTGATTCC | [1] |
| 53_CA5377 | cacggcgcgcctagcagcggCATTTATATTAACTACTTTTCTCCTATGG | [1] |
| 35_CA5377 | gtcagcggccgcatccctgcTAGTTTGTCTTGATACACATATACATATATATATA | [1] |
| 33_CA5377 | GAAAAATGGATGCCAAGTTG | [1] |
| 55_CA5148 | AGTAAACTTTTTGGTAGGGAACG |  |
| 53_CA5148 | cacggcgcgcctagcagcggGTTAAATTGAATTGAATTGAATTGC |  |
| 35_CA5148 | gtcagcggccgcatccctgcAACAATTCTCCATCATTGGATG |  |
| 33_CA5148 | GAAGGTGAAAATGAGAATGCAG |  |
| Mkc1_5’5’ | GCCGGTCCTACAGAAAGCCG |  |
| Mkc1_5’3’ | cacggcgcgcctagcagcggGATCCATTATGGAAATTGGTTC |  |
| Mkc1_3’5’ | gtcagcggccgcatccctgcCACACAACCACGTTATCCC |  |
| Mkc1_3’3’ | CAGCTCCACACAACTAGCATG |  |
| 55_CA2834 | TCTTCCAATGAATGGGTAGACC |  |
| 53_CA2834 | cacggcgcgcctagcagcggCATTTTTTCTTCGGTTGGTTTG |  |
| 35_CA2834 | gtcagcggccgcatccctgcTGAATGGCAAATAATGTAGATAGAAG |  |
| 33_CA2834 | TCTCAATATGTCAAACCATGTGG |  |
| 55_CaTPK1 | TCACACATTTATCCGTTTTGACAG |  |
| 53_CaTPK1 | cacggcgcgcctagcagcggCATCCTGGTTTTGGTTGATAAAAC |  |
| 35_CaTPK1 | ATGTAAAAATAAAATTGTATTTATTGGC |  |
| 33_CaTPK1 | ATTCTCGCTATTATGTTTGATTCC |  |
| 55_CaTPK2 | GCTATTCCATACATTGTTAAAAGTCG |  |
| 53_CaTPK2 | cacggcgcgcctagcagcggACCATCAAAAAGGGGGAAGG |  |
| 35_CaTPK2 | gtcagcggccgcatccctgcTGAATTGAGAGGAGATGAACTTTC |  |
| 33_CaTPK2 | CATGTCAAGGGTATTGTTAGTGTG |  |
| 55_CaSNT1 | CTTCTTTCCTCCCTTCACTC |  |
| 53_CaSNT1 | cacggcgcgcctagcagcggCATATTATGATAAAATTAATTATTTTGACAG |  |
| 35_CaSNT1 | gtcagcggccgcatccctgcCATGAGTTTGTTAAATTAGTACATATGTAG |  |
| 33_CaSNT1 | GGTAAAGGATCATCATTTCAAGG |  |
| 55_CaSIF2 | GCGACGTGAGAGTGAATTAGG |  |
| 53_CaSIF2 | cacggcgcgcctagcagcggCATAATGTAGCAAAAAACAAAGGG |  |
| 35_CaSIF2 | gtcagcggccgcatccctgcATCCACACAACATAAAGAACCC |  |
| 33_CaSIF2 | CAATCATTACCAATGGCAGC |  |
|  |  |  |
| **Cloning of the integration vector pRP53 and complementantion of *SET3*** | | |
| 55_RP10 | cccaagcttaccggtCCCACGTTAACAATTTCATCAAG |  |
| 53_RP10 | cgcggatccgctagcctcgaggggcccGCCATGTTGTACTTGAGTTGGTG |  |
| 35_RP10 | gtcggagctccgacTAAGGATAATGATAACTGAAGAGAAG |  |
| 33_RP10 | gctaccgcggtagcaccggtCTCGACTATTACTCATTGATAAAGAC |  |
| INT5_CA7221 | ctagctagctagCTGCAAAACTACAAGCCACAACC |  |
| INT3_CA7221 | gctagggccctagcGAACCCAATGTACCACATCAAGG |  |
|  |  |  |
| **Quantitative Real-time PCR** | | |
| RT5_ECE1 | TGCCATTTGTTGTCAGAGCTG |  |
| RT3_ECE1 | TAGCTTGTTGAACAGTTTCCAGG |  |
| RT5_HWP1 | GCTGGTTCAGAATCATCCATGC |  |
| RT3_HWP1 | AAGGTTCAGTGGCAGGAGCTG |  |
| RT5_SOD5 | TTGATTTGAAAGGATTGCCCTC |  |
| RT3_SOD5 | AGCAGCAGGAGTTGCAGCTC |  |
| RT5_RBT2 | CCGTCACCGAATCCAACACC |  |
| RT3_RBT2 | AACGGTAATGACGAACAACCCAG |  |
| RT5_DDR48 | GCTCCTCAAACACTGACAGTTACG |  |
| RT3_DDR48 | ACCTCTTCTGTTTGAGGAACCG |  |
| RT5_FRE2 | CACTCAACCTCAAGATTACAATGGC |  |
| RT3_FRE2 | GCAGGATGTCCACACGTAACG |  |
| RT5_RIP1 | TGCTGACAGAGTCAAGAAACC |  |
| RT3_RIP1 | GAACCAACCACCGAAATCAC |  |
| NRT5_CA7221 | TACCTTGGGAATGGGATGTGG |  |
| NRT3_CA7221 | CCGTTGATTCAACCTTTCCTCC |  |
| RT5_CA5377 | TTCCCAGGTACAGGGTCAGTTG |  |
| RT3_CA5377 | GATTGACATCATTCAATACGCTCG |  |

* Lower case and upper case letters denote exogenous and endogenous sequences, respectively.

**SUPPLEMENTARY REFERENCES**

1. Hnisz D, Schwarzmuller T, Kuchler K (2009) Transcriptional loops meet chromatin: a dual-layer network controls white-opaque switching in *Candida albicans*. Mol Microbiol 74: 1-15.
