# Supplementary material for: The Set3/Hos2 Histone Deacetylase Complex Attenuates cAMP/PKA Signaling to Regulate Morphogenesis and Virulence of Candida albicans
Source: PLoS Pathog. 2010 May 13;6(5):e1000889. doi: 10.1371/journal.ppat.1000889 (PMC2869326; doi:10.1371/journal.ppat.1000889)
Supplement: Table S3 — Plasmids used in this study. (0.04 MB DOC) [file ppat.1000889.s008.doc]

**Supplementary Table 3. Plasmids used in this study**

| **Name** | **Parent** | **Target locus** | **Fragment*** | **Reference** |
| --- | --- | --- | --- | --- |
| pSFS2A | - | - |  | [1] |
| pDH102 | pSFS2A | *HOS2* |  | [1] |
| pDH104 | pSFS2A | *SET3* |  | [1] |
| pDH112 | pSFS2A | *SET3* |  | [1] |
| pAG36 | - | - |  | [2] |
| pRP53 | pAG36 | *RP10* | RP10 upstream and downstream (55+53 and 35-33) | This study |
| P7221 | pRP53 | *RP10* | *SET3* ORF+promoter | This study |

**SUPPLEMENTARY REFERENCES**

1. Hnisz D, Schwarzmuller T, Kuchler K (2009) Transcriptional loops meet chromatin: a dual-layer network controls white-opaque switching in *Candida albicans*. Mol Microbiol 74: 1-15.

2. Goldstein AL, McCusker JH (1999) Three new dominant drug resistance cassettes for gene disruption in *Saccharomyces cerevisiae*. Yeast 15: 1541-1553.
